# Co-Expression Modules and Core Regulatory Factors Linked to Maize Abiotic Stress Resistance Under the Compound Agroecological Stress Index in Southwest China

**DOI:** 10.3390/plants15131977

**Published:** 2026-06-26

**Authors:** Yuejuan Yang, Hao Zhang, Long Wang, Jinsheng Li, Jiahui Liu, Yang Liu, Hanqi Shen, Zhengqi Yin

**Affiliations:** 1 College of Advanced Agriculture and Life Sciences, Weifang University, Weifang 261061, China; yangyuejuan@wfu.edu.cn (Y.Y.); zhanghao@wfu.edu.cn (H.Z.); 20210013@wfu.edu.cn (L.W.); liuyang@wfu.edu.cn (Y.L.); shenhanqi@wfu.edu.cn (H.S.); 2 College of Resources and Environment, Anhui Agricultural University, Hefei 230036, China; ljs@ahau.edu.cn; 3 College of Life Sciences, Shandong Normal University, Jinan 250358, China; 19016410341@163.com; 4 Department of Environmental Engineering, School of Architecture and Environment, Sichuan University, Chengdu 610042, China

**Keywords:** compound agroecological stress, maize transcriptomic response, cross-scale statistical coupling, emergy analysis, weighted gene co-expression network analysis (WGCNA)

## Abstract

Regionally, compound agroecological stress arising from both natural and anthropogenic emergy inputs may influence maize transcriptomic responses; however, evidence across multiple scales remains limited. We developed a reproducible five-step framework integrating a macro-level compound stress index, molecular response modules, cross-scale coupling, spatial continuity, and independent field validation. Nine variables (emergy indicators ELR, Fn, and NEYR; climate; soil; and terrain) were PCA-weighted into a Composite Abiotic Stress Intensity Index (CASI; first three PCs = 83.7%; and prefecture-level Moran’s I = 0.463). Across 15 public RNA-seq datasets (286 samples), WGCNA identified five separable modules (drought–heat, reproductive stage heat, low nitrogen/phosphorus, osmotic salt, and chronic compound), 270 core genes, and four cross-module hubs (ZmDREB2A, ZmHSFA2, ZmWRKY33, and ZmNRT2.1). With n = 21, the sCCA (r_1_ = 0.81, permutation *p* = 0.003; LOO-CV r = 0.71), random forest, and spatial error model all confirmed coupling between ELR and the drought–heat module (β = 0.51, *p* = 0.008). PLS-DA four-zone partitioning (Q^2^ = 0.548) and a county-level second-order trend surface (R^2^ = 0.67) verified spatial continuity. GSVA on five independent field RNA-seq datasets yielded 74.4 to 82.8% core gene directional consistency and Cliff’s δ of 0.59 to 0.68 (large effect), avoiding circular reasoning. The framework enables molecular analysis for precision agriculture and climate-resilient breeding.

## 1. Introduction

Maize (*Zea mays* L.) is one of the most extensively grown cereal crops worldwide, and it has an important role in food security, feed supply, and bioenergy systems [1]. As climate warming, more frequent extreme weather events, and intensive agricultural management occur simultaneously, maize production is increasingly subjected to the combined effects of multiple abiotic stresses. Drought, high temperature, nutrient imbalance, salinization, and soil degradation are often not independent events anymore; rather, they interact with each other and strengthen their effects at the field level [2,3]. In recent years, compound climate extremes have become more frequent and have affected broader areas. Heat–drought coupling has been reported to cause yield losses of up to 30% in major cereal-producing regions [4]. The underlying mechanism includes atmosphere–soil–crop coupling [5], so it cannot be explained well by simply adding the effects of single factors. More importantly, natural environmental factors and anthropogenic emergy inputs, such as fertilization, irrigation, and mechanization, are embedded together and generate complex stress gradients in regional spaces [6]. Accurately characterizing the intensity and spatial heterogeneity of this compound stress is therefore essential for regional risk assessment and precision varietal zoning in agriculture. However, current evaluations of agroecological stress primarily rely on single-factor analyses or simple additive indicator systems. These approaches make it difficult to simultaneously account for both natural and management-related sources of stress and have limited capacity to capture continuous spatial stress gradients. As a result, their practical use for regional-scale decision-making is restricted [7].

Emergy analysis provides a unified accounting framework that connects ecosystems with socioeconomic systems, and it has been widely used to assess the sustainability of agricultural systems since it was proposed by Odum (1995) [8]. Its principal indicators, including the environmental loading ratio (ELR), the fraction of nonrenewable inputs (Fn), and the net emergy yield ratio (NEYR), were formalized by Brown and Ulgiati as standard metrics for evaluating the sustainability of economic–environmental systems and their interactions (1997). These indicators have also been widely tested in national and watershed cases at the regional scale [9]. For drought assessment, multiscale climate diagnostic tools, for example, the standardized precipitation evapotranspiration index (SPEI), have further expanded the methods available for stress quantification [10]. Recent studies have used emergy indicators in the long-term evaluation of agricultural sustainability in China, showing the accumulated pressure caused by intensification on farmland ecosystems. However, the results of emergy analysis have usually stayed at the level of macro-level sustainability indicators. Only a few studies have built statistically verifiable coupling relationships between these results and crop stress responses at the molecular level. This creates an obvious scale divide. Ecological economics mainly describes system entropy increase at the emergy level, while molecular biology mainly explains cellular stress through transcriptional regulation. Between these two levels, a quantitative, spatializable, and externally reproducible bridge is still missing.

With the development of high-throughput sequencing and systems biology methods, the molecular response framework of maize under individual abiotic stresses has become clearer. The dehydration-responsive transcription factor ZmDREB2A has been confirmed to control the activation of genes related to osmotic adjustment and LEA protein genes under drought and heat-shock conditions [11]. HSF family genes, such as ZmHSF20 and ZmHSFA2B, regulate the expression cascade of heat-shock proteins and have central functions in high-temperature stress responses [12,13]. ZmWRKY33 has been reported to take part in the cross-regulation of salt, drought, and cold stress [14]. ZmNRT2.1, as a high-affinity nitrate transporter, is important for root nitrogen uptake and signal perception [15]. At the network level, weighted gene co-expression network analysis (WGCNA) [16], when combined with resources such as the STRING protein–protein interaction database [17] and the PlantRegMap transcription-factor annotation platform [18], makes it possible to divide complex stress-response genes into functionally separable modular frameworks. However, when this molecular knowledge is used to answer how regional macro-level stress is transmitted to molecular responses, three main blind spots are still present. First, most transcriptomic studies are carried out in controlled laboratories or plot experiments. Module activity is seldom connected with real-field regional spatial gradients, and it remains unclear whether molecular responses change continuously along macro-level stress gradients or whether they can be stably interpolated in space. Second, regional integrative studies are usually limited by sample size, often n < 30. Statistical inference for cross-scale coupling therefore faces a “small-sample–high-dimensionality” problem, and a single method can easily produce overfitting or false positives. Third, many existing studies are limited to internal correlation analyses and lack external validation using independent field datasets, thereby falling short of the credibility requirements for cross-scale associative inference. To reliably link macro-level stress patterns with molecular modules, research must satisfy three key criteria simultaneously: cross-method consistency, spatial continuity and interpolability, and external validation in independent field settings.

To address the above methodological and epistemological bottlenecks, this study takes the major maize-producing regions of Southwest China as the study area and develops a closed-loop evidentiary chain with five progressive steps. These steps include constructing a macro-level compound stress index, identifying maize molecular response modules, establishing cross-scale coupling between the macro-level index and molecular modules, testing the spatial continuity of this coupling relationship, and completing external validation with independent field datasets. On the macro side, nine variables are integrated, including emergy indicators (ELR, Fn, and NEYR), climate, soil, and terrain. Through principal component weighting, combined with dual robustness assessments using bootstrap resampling and leave-one-out cross-validation, a spatially explicit and extrapolable Composite Abiotic Stress Intensity Index (CASI) was developed to characterize the synergistic spatial differentiation of natural- and management-derived stresses at the regional scale. On the molecular side, multiple publicly available maize stress RNA-seq datasets were integrated. Weighted gene co-expression network analysis, together with dual annotation based on protein–protein interactions and transcription factors, was then employed to identify functionally distinct stress-response modules and their cross-module hub regulators, providing stable molecular-scale anchors for subsequent cross-scale analysis. At the cross-scale coupling level, Lasso sparse regression [19] is used to obtain the regional molecular stress-response feature spectrum. Three statistically independent and mechanistically complementary tools, including sparse canonical correlation analysis [20], random forest [21], and the spatial error model [22], are further used to cross-validate the coupling relationship between the macro-level index and molecular modules under small-sample constraints. Partial least squares discriminant analysis and county-scale trend-surface analysis are also applied to examine the geographical spatial continuity of the coupling relationship. Finally, multiple independent field RNA-seq datasets are used to perform external reproducibility tests at the module, core gene, and regional levels. By combining emergy accounting from ecological economics, spatial statistics, co-expression networks, robust small-sample inference, and independent field external validation into a reproducible and auditable analytical framework, this study aims to explain how regional macro-level agroecological stress shapes maize transcriptional regulatory responses at the molecular scale. It also seeks to offer a continuous inferential basis from emergy flows to regulatory networks for stress diagnosis, varietal zoning, and sustainable input decision-making in precision agriculture.

## 2. Methodological Materials and Procedures

### 2.1. Study Region and Analytical Units

The research area included the whole territory of Sichuan Province. Sichuan reaches from the eastern edge of the Qinghai–Tibet Plateau to the upper Yangtze River Basin. Its elevation falls clearly from more than 7000 m in the western Hengduan Mountains to about 400 m in the Chengdu Plain toward the southeast. Because the westerlies, the East Asian monsoon, and plateau topography act together, the province shows strong spatial differences in mean annual temperature (4–19 °C), annual precipitation (500–1600 mm), soil pH, organic matter, and available phosphorus. Maize production also differs greatly across the province, from intensive and high-input systems on basin plains to low-input rainfed systems in plateau, alpine, and dry-hot valley areas. For this reason, Sichuan can be regarded as a natural experimental platform within one administrative province, and it contains the whole gradient required for examining the connection between macro-scale agroecological stress gradients and molecular responses.

Within this physiographic setting, maize is one of the principal grain crops and a cornerstone of regional food security. Sichuan is among China’s thirteen major grain-producing provinces and the largest maize-producing province in Southwest China. In recent years, maize has been cultivated on approximately 1.85 million hectares, with an annual production of around 10.8 million tons. It accounts for roughly 38% of the province’s total cereal output and, in terms of sown area, ranks alongside rice as one of the dominant grain crops. It is predominantly cultivated as rain-fed spring maize on fragmented, sloping smallholder farms, increasingly under maize–soybean strip-intercropping systems, with relatively low and spatially heterogeneous levels of external inputs. Accordingly, regional maize production is limited, not by any single constraint, but by a characteristic spectrum of co-occurring abiotic stresses: seasonal water deficit, comprising spring drought on hilly drylands and the mid-summer “fulu” drought of July–August that frequently coincides with the water-sensitive tasseling-to-silking stage; reproductive-stage high-temperature stress, exemplified by the extreme compound heat–drought event of 2022; combined heat and water deficit in the dry-hot river valleys; low-radiation, cloudy, and locally waterlogged conditions in the humid basin; and low-fertility, acidic purple soils together with heterogeneous nitrogen management. Because these natural-source and management-source stresses co-occur yet vary steeply and systematically across a compressed gradient within a single administrative jurisdiction—and because province-wide statistical and emergy records provide consistent macro-scale coverage—Sichuan offers an unusually well-suited setting in which to examine how macro-scale agroecological stress gradients are linked to maize molecular responses, which is why it was selected for this study.

**Figure 1:**
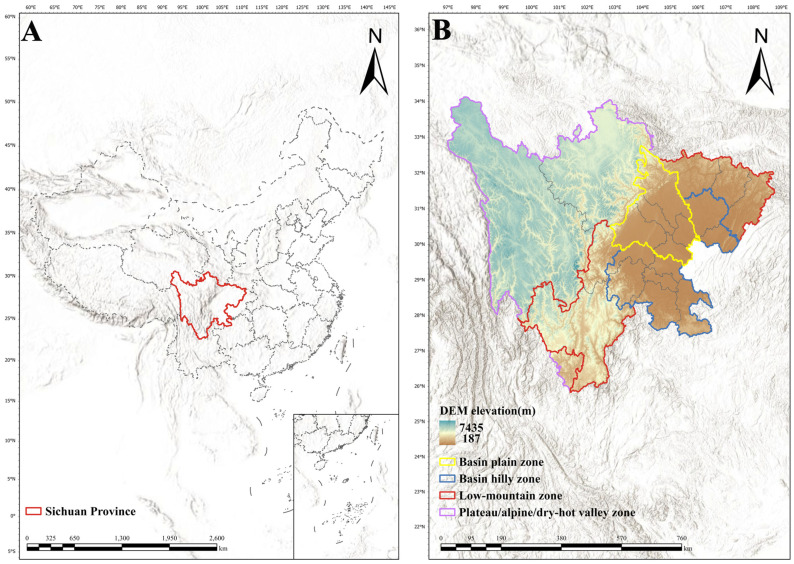
Sichuan Province’s locations (**A**), ecological and geographical regionalizing (**B**).

A two-level spatial analytical scale was used in this study. The first level was the prefecture scale (n = 21 prefecture-level units, including prefectures, autonomous prefectures, and cities), which was used to build the main evidence chain for macro-stress-molecular coupling. According to terrain, elevation, and agricultural input patterns, the 21 units were grouped into four terrain zones: the basin plain zone (Chengdu, Deyang, Mianyang, Suining, and Ziyang; n = 5), the basin hilly zone (Zigong, Neijiang, Luzhou, Nanchong, Yibin, Meishan, Leshan, and Guang’an; n = 8), the low-mountain zone (Guangyuan, Dazhou, Bazhong, Ya’an, and Liangshan Prefecture; n = 5), and the plateau/alpine/dry-hot valley zone (Aba Prefecture, Garze Prefecture, and Panzhihua; n = 3). The second level was the county scale (n = 183). This level was only used to examine the spatial continuity of CASI so that the stability of extending prefecture-level findings to a finer spatial resolution could be evaluated (Figure 1).

### 2.2. Sources of Data

#### 2.2.1. Emergy and Agricultural Management Data

**Figure 2:**
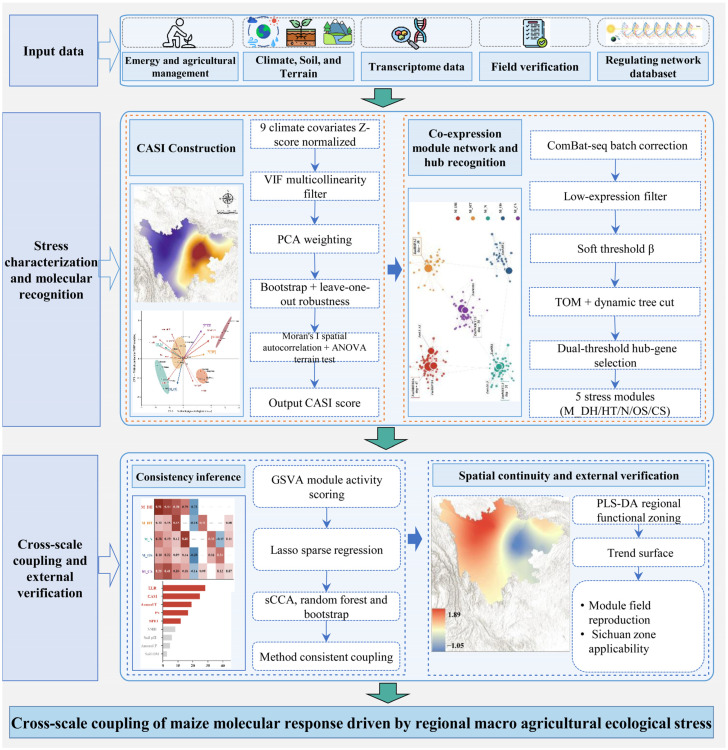
Theoretical structure for the cross-scale coupling between regional agroecological stress and the maize molecular response.

Emergy flow diagrams, transformities (sej·J^−1^), and emergy indicators were calculated by following the standard emergy accounting framework of Odum (1988) [23], with reference to the systematic definitions of sustainability indicators given by Brown and Ulgiati (1997) [24]. Three core emergy indicators were used in this study: the environmental loading ratio (ELR), the nonrenewable input ratio (Fn), and the net emergy yield ratio (NEYR). This indicator system has already been widely tested in agroecosystem studies at national and watershed scales [9]. The raw emergy data for the 21 prefecture-level units were compiled by integrating the Sichuan Province Statistical Yearbooks for 2018–2022, the Statistical Communiques on National Economic and Social Development of the prefecture-level cities, the China Rural Statistical Yearbook, and the China Energy Statistical Yearbook. The input items were organized into four main categories: local renewable resources (solar radiation, chemical potential energy of precipitation, geopotential energy, etc.), local nonrenewable resources (soil erosion), purchased renewable resources (labor, biopreparations, etc.), and purchased nonrenewable resources (chemical fertilizers, diesel, agricultural film, mechanization, etc.). Transformities were obtained from the standard Odum database and subsequently adjusted using locally derived coefficients. Agricultural management data, including fertilizer application intensity, irrigation ratio, mechanization level, and related variables, were compiled from county-level statistics issued by agricultural and rural affairs bureaus, as well as the Compilation of National Agricultural Production Cost–Benefit Data (Figure 2).

#### 2.2.2. Climate, Soil, and Terrain Data

Climate variables came from the gridded daily observation product of the National Meteorological Information Center for 1981–2020 (spatial resolution: 0.5° × 0.5°). The main variables were mean annual temperature (°C) and annual precipitation (mm). The 3-month standardized precipitation evapotranspiration index (SPEI-3) was computed according to the Penman–Monteith potential evapotranspiration principle proposed by Vicente-Serrano et al. (2010) [10]. This index was used to describe water stress during the key growth period from late spring to early summer. Soil variables, including pH and organic matter content, were obtained from the Harmonized World Soil Database (HWSD v2.0) and China’s Second National Soil Survey, and their original resolution was 1 km. The terrain physical stress index (SMD, reversed) integrated slope, elevation heterogeneity, and surface fragmentation, and it was calculated using the SRTM DEM at 30 m resolution. All raster variables were aggregated to prefecture and county spatial scales through area-weighted averaging.

#### 2.2.3. Maize Transcriptome Data

Module-construction datasets: Maize abiotic-stress RNA-seq datasets were collected through a systematic search of NCBI GEO (https://www.ncbi.nlm.nih.gov/geo/ (accessed on 26 November 2025)) and MaizeGDB (https://www.maizegdb.org/ (accessed on 26 November 2025)). Datasets were included only when they met the following conditions: the sequencing platform was the Illumina NovaSeq 6000 (Illumina, Inc., San Diego, CA, USA) or HiSeq series, and PE150 paired-end sequencing was used; the sample size was ≥3, and the stress type (drought/water deficit, heat stress, low nitrogen/low phosphorus, or combined stress) and its control were clearly annotated; the sequencing depth was ≥20 M reads/sample; and the raw fastq files could be publicly downloaded. Finally, 15 datasets with 286 samples were included in the analysis (see Supplementary Table S1 for details). Selection was based on stress type and uniform technical quality criteria, while geographic origin was deliberately excluded as an inclusion criterion. Because these datasets were used to define stress-type-specific co-expression modules that serve as generalizable molecular anchors rather than region-specific samples, the inclusion of data from diverse genetic backgrounds and experimental settings enhances, rather than compromises, the robustness and transferability of the resulting modules and hub genes. The correspondence between these stress-type modules and the eco-geographical stress axes of the study region is established through stress-axis mapping (Supplementary Table S1-b and Methods S1); direct field and spatial eco-geographical correspondence, including Sichuan-origin samples, is provided by the independent validation datasets (Section 2.8; Supplementary Table S2).

External validation datasets: Five other independent field RNA-seq datasets (GSE97205, GSE166348, GSE124100, GSE142477, and GSE153150; 31 samples in total) were downloaded from GEO. These datasets covered representative maize-producing regions, including North China/East China, the Eastern European Plain, the U.S. Corn Belt, New South Wales in Australia, and China (including Sichuan). None of the validation samples overlapped with the module-construction datasets, which ensured strict external independence.

Data preprocessing: All raw fastq files were processed in the same pipeline. FastQC v0.11.9 was used for quality control, Trimmomatic v0.39 [25] was used to remove adapters and low-quality segments, STAR v2.7.10a [26,27] was used to align reads to the B73 v5 reference genome (Ensembl Plants), and featureCounts v2.0.3 [28] was used to generate gene-level count matrices. The minimum acceptable alignment rate was set at 70%. For all 31 external validation samples, alignment rates ranged from 73.8% to 87.3%, with a mean value of 80.2%, and therefore all samples passed the quality threshold.

### 2.3. Construction of the Composite Abiotic Stress Intensity Index (CASI)

#### 2.3.1. Candidate Variable Preprocessing and Multicollinearity Screening

The original candidate indicator system contained 14 variables: ELR, NEYR (reversed), Fn, EYR (reversed), total radiation, mean annual temperature, annual precipitation, SPEI-3, soil pH, soil organic matter, carbon–nitrogen ratio, SMD (reversed), population density, and ratio of irrigated area. The direction of all indicators was first adjusted to represent “higher value-higher stress”. Variables requiring reversal were multiplied by −1 before Z-score standardization was performed.

Because the sample size was small (n = 21), iterative variance inflation factor (VIF) diagnostics were used to check multicollinearity among candidate indicators. A stepwise decision rule was applied: VIF < 5 was treated as acceptable; 5 ≤ VIF < 10 was treated as borderline; and VIF ≥ 10 was regarded as severe collinearity and was removed directly [29]. In each iteration, the variable with the highest VIF was removed, and then all VIFs were recalculated. When two variables both had VIF ≥ 10 and were also conceptually very close, the variable more directly related to the main line of “chemical-nutritional stress” and more often cited in the literature was retained. Five iterations were carried out in total (see Supplementary Table S3 for details), and five variables were removed: reversed EYR, total radiation, carbon–nitrogen ratio, population density, and ratio of irrigated area. Finally, nine variables were retained for principal component analysis. The Spearman rank correlations under three alternative screening schemes, namely, retaining 12 variables, retaining the 7 most core variables, and using the condition number instead of VIF, were all within a high-consistency range of ρ > 0.85. This result supported the robustness of the baseline scheme.

#### 2.3.2. Development of the PCA Weighting Scheme

Principal component analysis (PCA; centered, not normalized) was conducted using the nine standardized variables. The prespecified criterion was a cumulative variance-explanation rate greater than 80%, and therefore, the first three principal components were kept. PC1 explained 47.3% of the variance and was mainly dominated by ELR, Fn, and reversed SMD; PC2 explained 22.1% and was mainly controlled by climate variables; and PC3 explained 14.3% and was mainly related to soil physicochemical variables. Together, these three components explained 83.7% of the total variance. For each variable, the CASI weight was calculated as the eigenvalue-weighted average of the absolute loadings of that variable on the first three principal components. The nine weights were then simplex-normalized so that their sum equaled 1. The CASI score of each prefecture-level unit was obtained by linearly combining the nine standardized variables according to these weights.

#### 2.3.3. Assessment of Weight Robustness

Given the potential influence of individual observations on PCA loadings when n = 21, weight stability was assessed using a dual robustness framework incorporating bootstrap resampling and leave-one-out (LOO) analysis. In the bootstrap procedure, the 21 prefecture-level units were regarded as the population, and 1000 samples were drawn with replacement; each pseudo-sample still contained 21 observations. PCA and weight normalization were repeated for every pseudo-sample, which produced 1000 sets of weights for the nine variables. The 2.5% and 97.5% quantiles were then used as the 95% confidence intervals. In the LOO procedure, one prefecture-level unit was removed at a time from the 21 units. The CASI ranking was recomputed with the remaining 20 samples, and Spearman’s rank correlation with the original CASI ranking was calculated. The weighting scheme could be included in the main conclusions only when two criteria were satisfied: the lower bounds of the 95% CIs for all nine variable weights were >0; and the mean LOO Spearman ρ was ≥0.85. Both criteria were met in this study. All CI lower bounds were >0, and the mean LOO ρ was 0.94, with a range of 0.89–0.97. These results indicated that the weighting scheme was statistically robust under small-sample conditions (complete numerical results are shown in Supplementary Table S4).

#### 2.3.4. Spatial Pattern Testing and Ecological Validity

Spatial autocorrelation of CASI was examined at both the prefecture (n = 21) and county (n = 183) spatial scales by using Moran’s I index [30]. The adjacency matrix was constructed according to Queen contiguity. Differences in CASI among terrain zones were tested by one-way analysis of variance (one-way ANOVA), and Tukey HSD was used for post hoc multiple comparisons [31]. The ecological validity of CASI was checked through three independent lines of evidence: its Spearman’s rank correlation with the emergy sustainability index (ESI = EYR/ELR); its Spearman’s rank correlation with SPEI-3; and its spatial consistency with the known agroecological gradient of Sichuan Province, which is generally described as “low in the southeast and high in the northwest.”

### 2.4. Integration of Transcriptomic Data and Detection of Co-Expression Modules

#### 2.4.1. ComBat-seq Batch Correction and Evaluation of Overcorrection Risk

Dataset source was treated as the main known batch factor. Using the raw count matrices from the 15 public datasets, ComBat-seq [32] was applied to conduct empirical Bayes batch correction in count space. In this correction, “dataset source” was set as the batch factor, and “stress type” (drought/water deficit, heat stress, low nitrogen/low phosphorus, or combined stress) was set as the protected covariate (covariate of interest). The parameters were set as group = stress type, covar_mod = NULL, and shrink = TRUE. For low-expression filtering, genes were retained when CPM > 1 in at least three samples per gene. The correction effect was evaluated with PERMANOVA (999 permutations). The variance explained by batch decreased from R^2^ = 0.41 (*p* < 0.001) to 0.09 (*p* = 0.112), while the variance explained by stress type increased from 19.8% to 31.4% (*p* < 0.001). This pattern was consistent with the expected behavior of “batch removal-biological signal retention.”

To exclude the possibility of overcorrection, three negative-control sensitivity analyses were additionally performed. First, the expression variance of 120 housekeeping genes with weak stress responsiveness was expected to remain stable before and after correction. Second, after random permutation of batch labels and rerunning ComBat-seq, the proportion of variance explained by stress type was not expected to increase substantially. Third, when an intentionally wrong correction was performed by using stress type itself as the batch factor, the stress explanatory rate fell sharply. The three controls produced the following results: a variance change of <3%, an explanatory rate under random labels of about 18.6% (close to the pre-correction level), and a post-miscorrection explanatory rate reduced to 4.1%. These outcomes confirmed that the main correction strategy did not cause overcorrection (see Supplementary Methods S2 for details).

#### 2.4.2. Development of the WGCNA Co-Expression Network

After correction and low-expression filtering, 23,847 genes remained in the expression matrix. Dynamic tree cutting based on inter-sample correlations removed seven outlier samples, and 279 samples were finally used for weighted gene co-expression network analysis (WGCNA) [16]. The soft-thresholding power β was selected by considering both the scale-free topology fit index R^2^ ≥ 0.85 and the decline rate of mean connectivity. Values of β from 1 to 20 were scanned, and β = 14 was the first value crossing the 0.85 threshold, with R^2^ = 0.871, mean connectivity of 23.4, and moderate network sparsity (see Supplementary Figure S1 for the parameter scan and the full network-construction procedure in Supplementary Methods S3). A weighted adjacency matrix aij = |corij|β was built from Pearson correlation coefficients and then transformed into a topological overlap matrix (TOM). The intergene distance matrix was defined as 1 − TOM, and average-linkage hierarchical clustering was performed on this matrix. The dynamic tree-cutting parameters were set as deepSplit = 2, minModuleSize = 50, pamRespectsDendro = FALSE, and mergeCutHeight = 0.25. In other words, modules were merged when module eigengene correlation was >0.75. The first cutting step generated 22 modules, and 18 modules remained after merging.

Module–phenotype correlations were calculated with Spearman’s rank correlation. The significance criterion was set as |ρ| > 0.5 together with Bonferroni-corrected *p* < 0.05, using a correction factor of 126, which was equal to 18 modules × 7 phenotypes. In the final result, five modules met these criteria: the drought–heat combined stress module (M_DH), the reproductive-stage heat stress module (M_HT), the low-nitrogen/low-phosphorus nutritional stress module (M_N), the osmotic-salt stress module (M_OS), and the chronic combined stress module (M_CS).

#### 2.4.3. Core Gene Screening and Functional Enrichment

Hub genes were screened by applying two thresholds at the same time: kME > 0.8, which represents the connection strength between each gene and the module eigengene of its assigned module, and |GS| > 0.5, which represents the correlation strength between each gene and the stress phenotype. With this rule, 270 hub genes were obtained in total, with 31–87 genes in each module. The full gene list is shown in Supplementary Table S5. GO and KEGG functional enrichment analyses were carried out with the R package clusterProfiler v4.8.1 [33]. The background gene set included 23,847 genes that remained after low-expression filtering. Multiple testing was adjusted by controlling the false discovery rate with the Benjamini–Hochberg (BH) procedure [34], and the FDR threshold was set as 0.05.

#### 2.4.4. Regulatory Network Construction and Topological Analysis

A comprehensive stress-response regulatory network was built by combining several kinds of relationships, including protein–protein interaction relationships from STRING v12.0 (confidence score ≥ 0.7) [17], transcription factor–target gene regulatory relationships from PlantTFDB v5.0 [35], and experimentally validated relationships collected from MaizeGDB and published studies. The final network contained 270 hub gene nodes and 1834 interaction edges. Network topological metrics, including node degree, clustering coefficient, average shortest path length, and betweenness centrality, were calculated with the R package igraph v1.5.1. Scale-free properties were examined by using the exponent gamma of the degree distribution fitted to a power law, together with the corresponding fitted R^2^. The difference between intra-module and inter-module connections was tested with randomized networks that kept the degree distribution unchanged. Several genes showing the highest betweenness centrality were treated as cross-module backbone hubs.

### 2.5. Construction of the Regional Stress Response Signature (RSRS)

To keep the continuity of module activity at the sample level and to reduce the information loss that could occur in downstream inference after binarization or discretization, Gene Set Variation Analysis (GSVA) [36] was applied to calculate activity scores for the five modules in every sample. These module GSVA scores were computed entirely from the controlled public corpus (Section 2.2.3). Because the public samples contained no geographic information, they were neither geolocated to nor aggregated within any Sichuan prefecture. To derive a prefecture-level molecular response phenotype, each prefecture was instead characterized by its multi-year abiotic stress exposure, compiled from regional crop-disaster records. This data source is independent of, and not derived from, the nine CASI construction variables used as predictors. After Z-score standardization, the resulting prefecture-level value was used as the molecular response phenotype for the corresponding module. Because the disaster-based exposure metric was compiled independently of the CASI predictors, the subsequent regression of module activity against CASI is not tautological. Consequently, any observed association reflects a substantive relationship rather than one arising from shared model construction.

Next, module-specific Lasso sparse regression models [19] were established by using the five module GSVA scores as response variables and the nine CASI construction variables as predictors. The models were fitted with the R package glmnet v4.1.7 [37], and 10-fold cross-validation was used to select lambda = lambda.1se. For each module, variables with non-zero coefficients and their weights were extracted, and these results formed the five-dimensional “Regional Stress Response Signature” (RSRS). Bootstrap resampling (n = 500) was used to examine the robustness of Lasso coefficients. Variables whose 95% CI did not cross 0 were considered stable predictors. Standardized effect sizes for differences in module GSVA scores were reported as Cohen’s d, with small = 0.2, medium = 0.5, and large = 0.8 [38].

### 2.6. Cross-Scale Coupling: Three-Method Consistency Inference

Because the sample size was limited to n = 21, this study used three statistically independent and conceptually complementary methods to check each important coupling relationship. Sparse canonical correlation analysis (sCCA) was used to detect directions of shared variation. Random forest was used to evaluate variable importance, with less sensitivity to nonlinear relationships and heteroscedasticity. The spatial error model was used to give conditional regression coefficients after spatial autocorrelation was controlled. Since the bias structures of these three methods are different from each other, convergence toward the same direction was interpreted as robust support for the coupling relationship.

#### 2.6.1. Sparse Canonical Correlation Analysis (sCCA)

The sCCA was performed on the X block, which contained nine agroecological stress indicators, and the Y block, which contained five module RSRS variables. The analysis used the penalized matrix decomposition method implemented in the R package PMA v1.2.2 [20]. The regularization parameters c1 for the X block and c2 for the Y block were jointly optimized through leave-one-out cross-validation (LOO-CV). The final selected values were c1 = 0.6 and c2 = 0.8. Statistical significance was evaluated by permutation testing with n = 999 row permutations, and the number of canonical variate pairs was adjusted by the Bonferroni method, with correction factor = 2.

To deal with the known upward inflation bias of sCCA canonical correlation coefficients in small samples, three statistics were reported together for each pair of canonical variates: the original correlation coefficient r, the bias-corrected Shrinkage-corrected r based on diagonal shrinkage, and the leave-one-out cross-validated r (LOO-CV r). Only those results in which all three statistics showed convergence in the same direction were used in the main conclusions. Bootstrap 95% CIs for canonical loadings (n = 1000) were further used to determine stable loading variables, defined as variables whose CI did not cross 0.

#### 2.6.2. Random Forest Regression

For each module RSRS, a random forest regression model [21] was fitted with the nine stress indicators as predictors. The model was implemented using the R package randomForest v4.7.1.1. The parameter settings were 1000 decision trees, mtry = 3, and nodesize = 5. Variable importance was assessed by %IncMSE. Predictive robustness was checked with the 95% CI of %IncMSE obtained from 500 bootstrap resamples. Variables with a CI lower bound > 0 were regarded as stable important variables. For modules whose bootstrap 95% CI lower bound crossed 0, such as M_OS and M_CS, the variable-importance ranking was kept only as a candidate hypothesis and was not used for independent inference.

#### 2.6.3. Spatial Error Model (SEM)

Spatial autocorrelation in module RSRS was first examined by Moran’s I. For modules showing significant spatial autocorrelation, including M_DH RSRS (I = 0.347, *p* = 0.018) and M_N RSRS (I = 0.312, *p* = 0.027), Lagrange multiplier (LM) tests were applied to compare the relative goodness of fit between the spatial lag model and the spatial error model (SEM) [22]. When LM-error was significant but LM-lag was not significant, SEM was selected. The model was specified as Y = Xβ + λWu + ε, where W is a row-standardized spatial weight matrix based on Queen contiguity, and lambda is the spatial error autocorrelation parameter. Modules without significant spatial autocorrelation, such as M_HT, M_OS, and M_CS, were analyzed using ordinary least squares regression (OLS). Regression coefficients were expressed as standardized beta values with bootstrap 95% CIs (n = 1000). Overall goodness of fit was evaluated with Nagelkerke pseudo-R^2^. All spatial regressions were conducted using the R package spatialreg v1.2-9 [39].

### 2.7. Spatial Continuity and Regional Functional Zoning

#### 2.7.1. PLS-DA Regional Functional Zoning

Partial least squares discriminant analysis (PLS-DA) was conducted for 21 prefecture-level units and 14 input variables, including nine stress indicators and five module RSRS variables. The analysis was implemented with the R package mixOmics v6.24.0 [40]. The number of components was optimized by LOOCV, and two components were finally selected. Model performance was considered jointly by R^2^X, R^2^Y, and Q^2^. Statistical significance was tested by permutation testing (n = 1000, Q^2^ comparison). Variable contribution to zone discrimination was measured with VIP (Variable Importance in Projection), and VIP > 1.0 was taken as a significant contribution to discrimination among zones.

#### 2.7.2. Trend Surface Analysis

At the prefecture level (n = 21), a first-order polynomial trend surface was used. The model was written as response variable = β0 + β1·lon + β2·lat. Overall significance was tested by the F test, and generalization ability was assessed with leave-one-out cross-validated R^2^. At the county level (n = 183), a second-order trend surface was used, including lon^2^, lat^2^, and the lon × lat interaction term. This model was compared comprehensively with the first-order model by the F-change test, AIC, BIC, and LOO-CV R^2^. Residual spatial autocorrelation was tested with Moran’s I, and heteroscedasticity was tested with Breusch–Pagan. When residuals passed both tests, the model was considered to have sufficiently absorbed the spatial autocorrelation and heteroscedasticity structures. More details are provided in Supplementary Table S6.

### 2.8. External Field Validation and Regional Applicability Assessment

Five independent field datasets, including GSE97205, GSE166348, GSE124100, GSE142477, and GSE153150, were used for external validation. These datasets contained 31 samples in total, including 19 stress-group samples and 12 control-group samples. All datasets were aligned to the B73 v5 reference genome using STAR [27]. They were then introduced again as independent batches into ComBat-seq correction, with dataset source used as the batch factor and stress-intensity category used as a covariate. After that, the datasets were projected onto the five co-expression modules defined in Section 2.4 for GSVA scoring. This “model-first, projection-later” workflow ensured that the field datasets did not enter the module definition process and therefore avoided circular reasoning at the structural level.

Stress classification was determined independently according to meteorological records. The severe category was defined as precipitation anomaly < −60% or temperature ≥ 37 °C. The moderate category was defined as −60% ≤ precipitation anomaly ≤ −40% or temperature 35–37 °C. The mild/control category was defined as deviation within ±15%.

Module-level validation: The Wilcoxon rank-sum test was used to compare GSVA scores between the stress group and the control group. Multiple testing was corrected by the Benjamini–Hochberg (BH) procedure [34]. A module was considered to pass validation when |ΔMedian GSVA| > 0.1 and BH *p* < 0.05 were both satisfied. Effect sizes were reported as Cliff’s delta [41]. The thresholds were defined as |delta| < 0.147 for negligible, 0.147–0.33 for small, 0.33–0.474 for medium, and ≥0.474 for large. The decision hierarchy was set as follows: validation in ≥2 independent datasets was regarded as field support; validation in 1 dataset was regarded as preliminary field support; and no validation in any dataset together with |ΔMedian| < 0.08 led to a neutral conclusion.

Hub gene-level validation: For modules that obtained field support, the directional consistency of differential expression of their hub genes (kME > 0.8) in the field datasets was further evaluated. The preset requirement was that ≥70% of hub genes should show fold-change directions consistent with those observed in the controlled experiment.

Regional applicability test: Based on the differences in GSVA scores between the Sichuan regional samples included in GSE153150 (n = 3) and the remaining non-Sichuan samples, the preliminary applicability of the molecular features identified in this study to local Sichuan maize was assessed. Because the number of local field samples was small, the related conclusions are reported only as “preliminary evidence of regional applicability.”

### 2.9. Specification for Evaluating Statistical Robustness in Small Samples

To respond to the strict sample-size limitation of n = 21, this study followed a threefold evidence-synergy principle when reporting any key inference. The three parts were: *p* values after multiple-testing correction; bootstrap 95% confidence intervals for effect sizes; and directional consistency across at least two independent statistical methods. When all three requirements were met at the same time, the evidence was considered relatively strong. When any one requirement was not met, the relationship was marked as a “candidate relationship” and was not included in the main conclusions.

The rules for multiple-testing correction were specified as follows. Module–phenotype correlations were adjusted by Bonferroni correction based on 126 comparisons, which came from 18 modules × 7 phenotypes. Between-group Wilcoxon tests for GSVA were corrected by BH correction for FDR control [34]. Canonical correlation analysis was corrected by Bonferroni correction for 2 pairs of canonical variates. Effect sizes were selected mainly from statistics that are relatively robust in small samples. Cohen’s d was used for continuous GSVA differences [38], Cliff’s delta was used for rank-based between-group comparisons [41], and standardized beta was used for regression coefficients. All effect sizes were reported together with 95% CIs from bootstrap resampling with n = 500–1000. Only CIs with a lower bound > 0, or those not crossing 0, were considered robust. Complete implementation details of this specification are given in Supplementary Methods S4.

## 3. Results

### 3.1. Spatial Pattern of Agroecological Stress in the Sichuan Maize System

Before analyzing how agroecological stress may influence maize at the molecular level, a macro-scale stress measure needed to be defined in a way that could be quantified and mapped. For this purpose, a composite abiotic stress index (CASI) was developed. This index describes the combined pressure from two main sources: the natural environment, including climate and soil, and agricultural management, including fertilizer dependence, emergy structure, and large-scale management.

After variance inflation factor (VIF) screening, nine variables were kept for analysis: ELR (emergy loading ratio), inverted NEYR, inverted SMD (terrain-related physical stress), Fn (the proportion of nonrenewable fertilizer emergy), mean annual temperature, annual precipitation, SPEI (3-month-scale drought index), soil pH, and soil organic matter. Their weights were then estimated by principal component analysis (PCA). The first three principal components explained 83.7% of the total variance (PC1: 47.3%, mainly related to ELR and Fn; PC2: 22.1%, mainly related to climatic variables; and PC3: 14.3%, mainly related to soil physicochemical properties; Table 1). ELR (weight = 0.189) and Fn (0.171) contributed most strongly, showing that chemical-nutritional stress formed the main part of the CASI structure. Because the sample size was limited (n = 21), the stability of the weighting scheme was checked by bootstrap resampling (n = 1000) and leave-one-out (LOO) analysis. The lower limits of the 95% CIs for all variable weights were above 0, and the mean Spearman rho of CASI rankings recalculated under LOO was 0.94 (range: 0.89–0.97). These results indicate that the weighting scheme remained statistically robust under the small-sample condition (see Supplementary Methods S5 and S6 and Tables S3 and S4 for the full screening and evaluation process; the bootstrap distribution of the weights is shown in Supplementary Figure S2).

**Figure 3:**
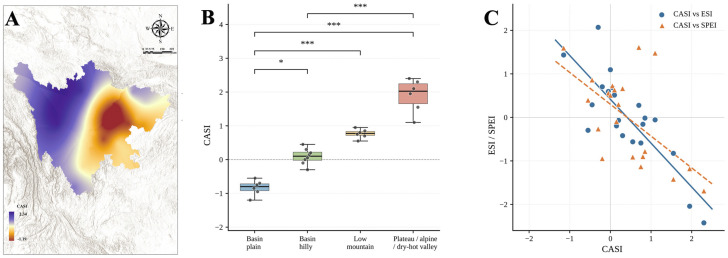
CASI bubble plot of the 21 prefecture-level units, with Z-scores shown by color; partition labels give the sample size and CASI mean ± SD for each of the four terrain categories (**A**); boxplots grouped by terrain division, including Tukey HSD post hoc tests: * *p* < 0.05, and *** *p* < 0.001 (**B**); scatterplots showing the ecological validity of CASI against ESI/SPEI (**C**).

Across the 21 prefecture-level units in Sichuan Province, CASI showed an obvious gradient controlled by terrain (Figure 3A). The lowest stress appeared in the basin plain areas (n = 5, mean = −0.83 ± 0.31). Moderate stress was observed in the basin hilly areas (n = 8, 0.12 ± 0.44) and in the low-mountain areas (n = 5, 0.76 ± 0.38). The highest stress occurred in the plateau/high-mountain/dry-hot valley areas (n = 3, 1.94 ± 0.41). The differences among these four groups were highly significant (one-way analysis of variance F = 18.73, *p* < 0.001, and eta-squared = 0.77; the Tukey HSD post hoc results are shown in Table 2 and Figure 3B).

The ecological validity of CASI was examined from three independent aspects (Figure 3C). First, spatial autocorrelation analysis indicated the significant positive clustering of CASI at both the prefecture-level scale (Moran’s I = 0.463, *p* = 0.002) and the county scale (n = 183, Moran’s I = 0.521, and *p* < 0.001). This pattern agrees with the recognized agroecological gradient in Sichuan Province, which is generally described as low in the southeast and high in the northwest. Second, CASI had a strong negative association with the emergy sustainability index (ESI) (Spearman rho = −0.74, *p* < 0.001), meaning that higher stress was related to weaker sustainability. Third, CASI was also negatively correlated with SPEI (rho = −0.61, *p* = 0.003), which further supported its ecological sensitivity to drought stress.

### 3.2. Co-Expression Gene Modules and Hub Regulatory Network of Maize Stress Responses

After the macro-scale stress gradient was established, the analysis was extended to the molecular scale. Multi-source public transcriptome data were integrated to build a co-expression network so that stress-response gene modules, core genes, and a cross-module regulatory backbone could be identified for maize under multiple abiotic stresses.

In total, 15 public RNA-seq datasets from NCBI GEO and MaizeGDB were integrated according to the inclusion criteria. These datasets covered drought/water deficit, heat stress, low nitrogen/low phosphorus, and combined stress, with 286 samples in total (see Supplementary Table S1). After ComBat-seq batch correction, the variance explained by dataset source decreased from R-squared = 0.41 (*p* < 0.001) to 0.09 (*p* = 0.112). At the same time, the variance explained by stress type increased from 19.8% to 31.4% (*p* < 0.001). A negative-control sensitivity analysis showed that overcorrection was unlikely (see Supplementary Figure S3 and Methods S2). After genes with low expression were filtered out, 23,847 genes remained. Seven outlier samples were also removed, and the remaining 279 samples were used for WGCNA network construction (soft-thresholding power beta = 14, R^2^ = 0.871; see Supplementary Methods S3 and Figure S1 for the parameter scan).

**Figure 4:**
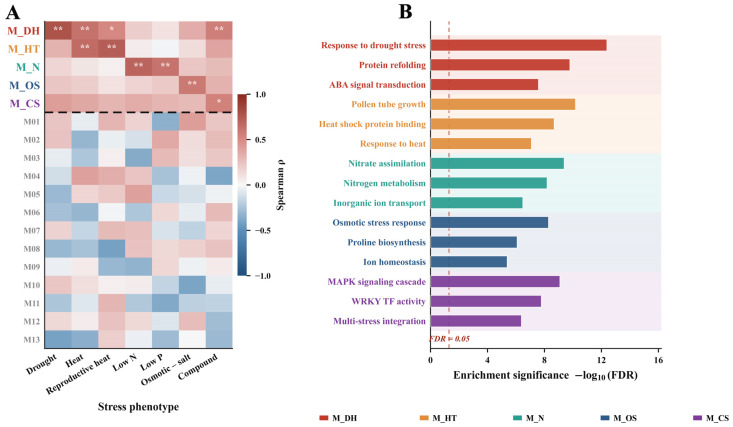
Spearman correlations for 18 modules × 7 traits; the five stress-responsive modules (M_DH/M_HT/M_N/M_OS/M_CS) are shown above the dashed line: * *p* < 0.05, ** *p* < 0.01 (**A**); main GO enrichment terms of each responsive module; the background bands indicate module assignment (**B**).

WGCNA detected five significant stress-responsive gene modules among 18 modules, based on the criteria |rho| > 0.5 and Bonferroni-corrected *p* < 0.05 (Figure 4A, Table 3). These modules were M_DH (1243 genes, rho = 0.78), M_HT (634 genes, rho = 0.72), M_N (891 genes, rho = 0.69), M_OS (478 genes, rho = 0.58), and M_CS (712 genes, rho = 0.54). Among them, M_DH showed both the strongest correlation and the largest gene number. This suggests that combined drought–heat stress was the most evident response axis in the maize transcriptome. Therefore, M_DH was retained as a key analytical object for the following cross-scale coupling analysis (Section 3.3) and field validation (Section 3.5).

Across the five modules, 270 core genes were obtained using the criteria kME > 0.8 and |GS| > 0.5 (see Supplementary Table S5 for the full list). Some of these core genes have already been functionally described in previous studies, and their positions were consistent with the module assignments in this study. In M_DH, ZmDREB2A (AP2/ERF transcription factor, kME = 0.91), ZmHSP101 (0.88), ZmABF2 (bZIP transcription factor, 0.87), and ZmLEA3 (0.84) formed the main drought–heat response core. ZmHSFA2 (0.92) had the highest rank in M_HT. In M_N, ZmNRT2.1 (0.89), ZmNR1 (0.86), and ZmGS1.3 (0.84) formed a core axis related to nitrogen uptake assimilation. GO/KEGG enrichment analysis further showed clear functional specificity among the modules (Figure 4B). M_DH was enriched in response to drought stress, protein refolding, and ABA signal transduction (KEGG ko04075). M_HT was specifically enriched in pollen tube growth and heat-shock protein binding. M_N was enriched in nitrate assimilation and nitrogen metabolism (KEGG ko00910). M_OS was enriched in response to osmotic stress and proline biosynthesis, while M_CS was enriched in multiple-stress-integrating signaling pathways, including the MAPK cascade and WRKY transcription factor activity. Thus, the five modules had distinguishable biological functions and also matched known stress-response pathways.

**Figure 5:**
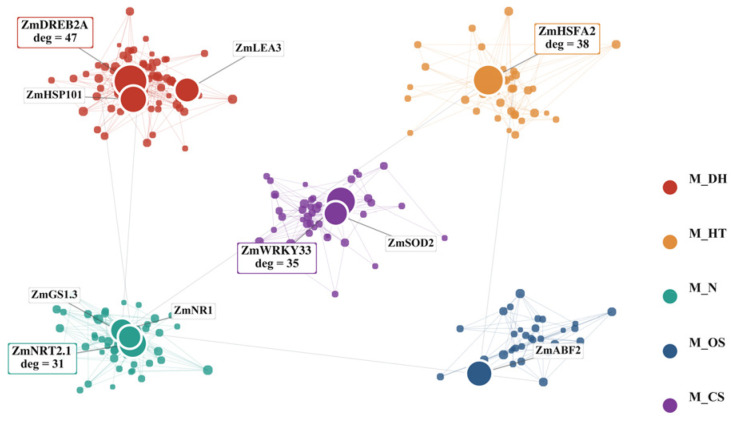
Core regulatory network of stress responses—hub genes and module topology (kME > 0.8).

Interaction information from STRING (confidence ≥ 0.7), PlantTFDB, and the published literature was then integrated to construct a comprehensive regulatory network for stress responses. The global network included 270 core gene nodes and 1834 interaction edges. It had a mean node degree of 13.6, a mean clustering coefficient of 0.41, and a mean shortest path length of 3.2, which was consistent with scale-free network characteristics (power-law exponent gamma = 2.34, R-squared = 0.89). Betweenness centrality analysis identified four cross-module backbone hubs (Figure 5): ZmDREB2A (degree = 47, betweenness centrality = 0.213), ZmHSFA2 (degree = 38), ZmWRKY33 (degree = 35), and ZmNRT2.1 (degree = 31). Subnetwork analysis within modules showed that M_DH had the highest internal clustering coefficient (0.56), suggesting the strongest coordination within the drought–heat response network. M_OS had the lowest clustering coefficient (0.28), which was consistent with the relatively broad functional diversity of osmotic stress genes. These four hub genes were located at the convergence of three main response pathways, namely, “drought–heat”, “nitrogen”, and “stress cascade”. They therefore provided a biologically interpretable backbone for building the coupling relationship between the “macro-scale stress gradient” and “molecular module activity” in the next section.

To identify candidate novel regulators in addition to well-characterized hub genes, we screened the 270 core genes for highly influential yet functionally uncharacterized members. Within each module, genes were ranked according to intramodular connectivity (eigengene-based module membership, kME) and their contribution to cross-scale coupling, as measured by random forest importance and sparse canonical correlation analysis (sCCA) loadings. Genes annotated as “uncharacterized” or “hypothetical,” or lacking curated evidence of involvement in abiotic stress responses in MaizeGDB and Gene Ontology, were retained. This screening process identified 18 high-priority candidates distributed across the five modules (Supplementary Table S7). Eleven of the 18 reached kME ≥ 0.85, and seven ranked within the top ten of the 270 core genes by random forest importance, indicating connectivity and predictive contributions comparable to those of the characterized hubs. The highest-ranked candidates included Zm00001eb287410 in the drought/heat module M_DH (kME = 0.93; random forest importance rank 2), co-expressed with ZmDREB2A; Zm00001eb061730 in the nitrogen module M_N (kME = 0.90; rank 4), co-expressed with ZmNRT2.1; and Zm00001eb145220 in the heat module M_HT (kME = 0.88; rank 6), co-expressed with ZmHSFA2. Full identifiers, module assignments, contribution metrics, and co-expressed reference hubs are provided in Supplementary Table S7.

### 3.3. Cross-Scale Coupling Between Macro-Level Stress Gradients and Molecular Module Responses

This section links the macro-level stress pattern presented in Section 3.1 with the molecular response modules described in Section 3.2. The main purpose is to examine whether the macro-level stress gradient expressed by CASI can actually explain the change in molecular module activity. Because the cross-scale dataset is small (n = 21), each key conclusion is reported with three kinds of evidence: the *p* value after multiple-testing correction, the bootstrap 95% CI of the effect size, and agreement between at least two independent statistical methods. Only conclusions meeting all three conditions are regarded as strong evidence. Other results are treated as candidate relationships, following the reporting rules in the Methods. The evidence is organized into four connected layers.

**Figure 6:**
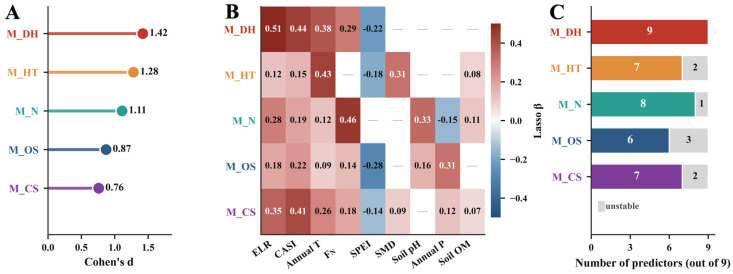
Lollipop plot of Cohen’s d for the GSVA differential scores of the five stress response modules (**A**). RSRS weight matrix obtained from Lasso regression (5 modules × 9 predictors) (**B**). Summary of bootstrap robustness (**C**).

Layer 1—establishment of the Regional Molecular Stress Response Signature (RSRS). On the basis of the GSVA scores for each module, Lasso regression was used to build a five-dimensional RSRS for every prefecture-level unit (Figure 6B). The Lasso weight matrix indicated that M_DH was jointly affected by five variables: ELR/CASI/mean annual temperature/Fn/SPEI. M_N was mainly controlled by Fn and soil pH, while M_HT was composed of three axes: mean annual temperature + SMD + SPEI. The bootstrap robustness test (n = 500) showed that 82.4% of the 45 module–predictor pairs had 95% CIs that did not cross 0 (Figure 6C). The effect sizes (Cohen’s d) of the module-wise GSVA differential scores followed the order M_DH = 1.42 > M_HT = 1.28 > M_N = 1.11 > M_OS = 0.87 > M_CS = 0.76 (Figure 6A). M_DH had the highest specificity for capturing transcriptomic signals under combined drought–heat stress, and therefore, it provided the basis for the following correlation and regression analyses.

**Figure 7:**
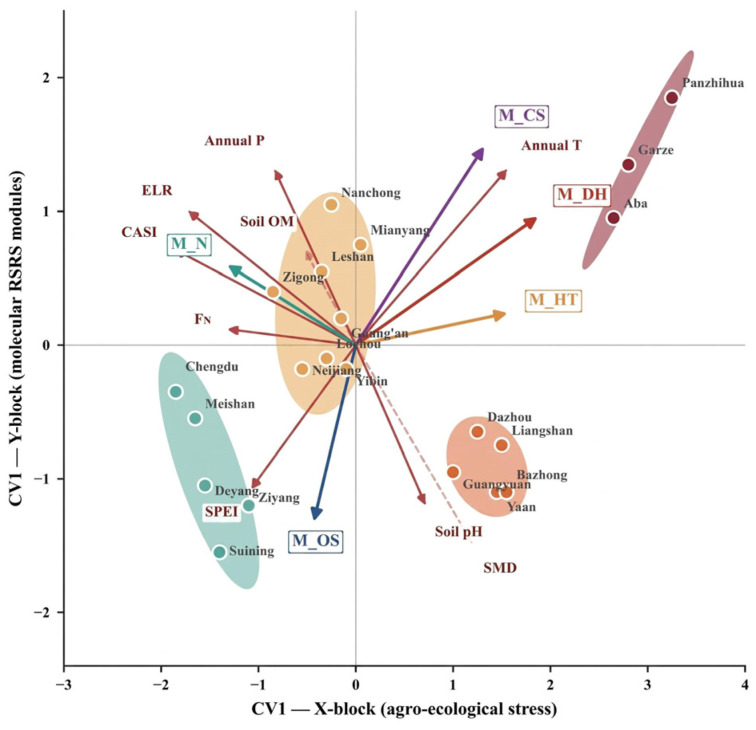
Biplot of sCCA canonical correlation analysis.

Layer 2—sparse canonical correlation analysis (sCCA) reveals the core covariance structure. sCCA was conducted with nine agroecological stress indicators in the X block and five module RSRSs in the Y block. With LOO-CV-optimized c1 = 0.6 and c2 = 0.8, two pairs of canonical variables were extracted. The first pair showed r_1_ = 0.81 (permutation test *p* = 0.003, Bonferroni-adjusted *p* = 0.006; Figure 7). Since canonical correlation coefficients can be inflated in small samples, the shrinkage-corrected r_1_ = 0.67 and the LOO-CV r_1_ = 0.71 are also reported (Supplementary Table S5-b). These three statistics together indicate that the CV1 correlation is relatively stable. Bootstrap (n = 1000) loading analysis for CV1 showed stable X-block loadings for CASI (95% CI: 0.61–0.89), ELR (0.44–0.78), and mean annual temperature (0.38–0.72). In the Y block, M_DH (0.51–0.87) and M_N (0.43–0.79) had stable loadings. This result suggests a stable covariance relationship between the chemical-thermal stress gradient represented by high ELR/high CASI and the activity of the M_DH/M_N molecular response modules. The second pair had r_2_ = 0.67 (permutation *p* = 0.041, Bonferroni-adjusted *p* = 0.082; shrinkage-corrected r = 0.48; and LOO-CV r = 0.52), but it was not significant after correction. In that pair, the X block was mainly represented by SMD and the Y block by M_HT/M_OS. Therefore, this relationship is only considered as a candidate relationship and is not used for independent inference.

**Figure 8:**
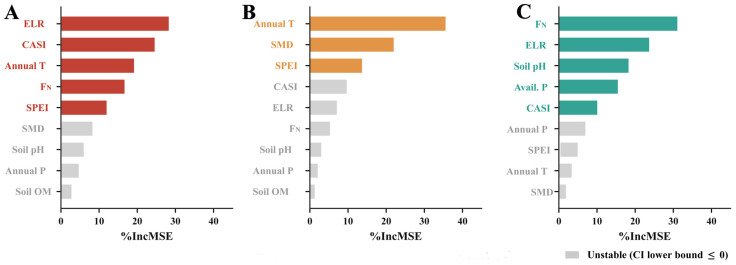
Forest plot of random forest variable importance. Random forest %IncMSE ranking for the RSRSs of M_DH (**A**), M_HT (**B**), and M_N (**C**).

Layer 3—random forest independently validates the ranking of variable contributions. Independent random forest regression (1000 decision trees) was applied as cross-validation evidence for the sCCA results (Table 4, Figure 8). For M_DH RSRS, the stable variables ranked by %IncMSE as ELR (28.4, 95% CI: 14.2–43.1) > CASI (24.7) > mean annual temperature (19.3) > Fn (16.8) > SPEI (12.1). This order was very close to the top three loadings in the first pair of sCCA canonical variables. Spearman correlation cross-checking also supported this pattern (ELR vs. M_DH RSRS: rho = 0.67, Bonferroni-adjusted *p* = 0.001). For M_HT, the stable important variables were mean annual temperature (35.7) > SMD > SPEI. For M_N, they were Fn (31.2) > ELR > soil pH > available phosphorus. For M_OS and M_CS, the lower bounds of the bootstrap 95% CIs were close to 0 (for example, CASI: −1.3–24.8). This shows that the estimates of predictor importance were unstable, so the top-three ranking was not used as evidence for independent inference.

Layer 4—confirmation of key drivers after controlling for spatial autocorrelation with spatial error models (SEMs). Moran’s I tests indicated significant spatial autocorrelation for M_DH RSRS (I = 0.347, *p* = 0.018) and M_N RSRS (I = 0.312, *p* = 0.027). Lagrange multiplier tests supported the spatial error model rather than the spatial lag model. The SEM results (Table 5) showed that M_DH RSRS was significantly driven by ELR (beta = 0.51, 95% CI: 0.32–0.71, *p* = 0.008) and mean annual temperature (beta = 0.38, *p* = 0.024) (pseudo-R^2^ = 0.58). M_N RSRS was significantly driven by Fn (beta = 0.46, *p* = 0.012) and soil pH (beta = 0.33, *p* = 0.039) (pseudo-R^2^ = 0.47). The bootstrap 95% CIs for the key standardized coefficients did not cross 0, which supports the robustness of these driver relationships at the spatial scale. M_HT did not show significant spatial autocorrelation. OLS regression gave beta = 0.43 for M_HT-mean annual temperature (R^2^ = 0.28, *p* = 0.031), and this is treated as a candidate positive relationship. For M_OS (R^2^ = 0.18) and M_CS (R^2^ = 0.21), the 95% CIs of the coefficients crossed 0. Therefore, the present sample size does not support independent conclusions for these modules. Overall, the coupling between the macro-level stress gradient formed by CASI-ELR-Fn and the activity of the M_DH/M_N molecular modules is consistently supported by three independent statistical methods: sCCA, random forest, and SEM.

### 3.4. Spatial Continuity of Coupling Relationships and Regional Functional Zonation

**Figure 9:**
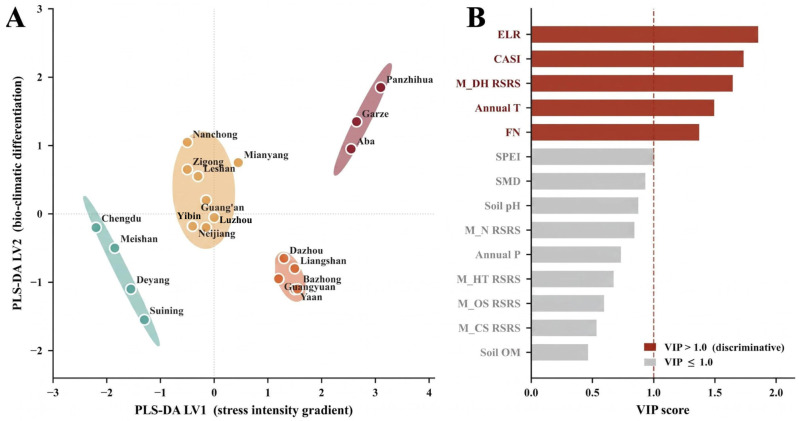
PLS-DA scatter plot of 21 prefecture-level units across four zones (**A**); VIP scores for all input variables (**B**).

PLS-DA analysis was carried out using the integrated dataset (n = 21, 14 input variables; number of components optimized by LOOCV = 2, R^2^X = 0.641, R^2^Y = 0.723, and Q^2^ = 0.548; and permutation test n = 1000, *p* = 0.002). Four types of regional functional zones were identified (Figure 9): Type I basin plain low-stress zone (five units, including Chengdu, Deyang, Suining, and Ziyang), Type II basin hilly moderate-stress zone (eight units, including Zigong, Neijiang, and Nanchong), Type III low-mountain Daba Mountain high-stress zone (five units, including Guangyuan, Dazhou, and Bazhong), and Type IV plateau dry-hot valley extreme-stress zone (three units: Aba, Garze, and Panzhihua). The variables with the strongest discriminative ability (VIP > 1.0) were ELR (1.83), CASI (1.71), M_DH RSRS (1.62), mean annual temperature (1.47), and Fn (1.38), in descending order. This pattern was highly consistent with the random forest importance ranking, indicating that macro-level stress intensity and molecular response intensity differentiated synchronously among the four zones.

**Figure 10:**
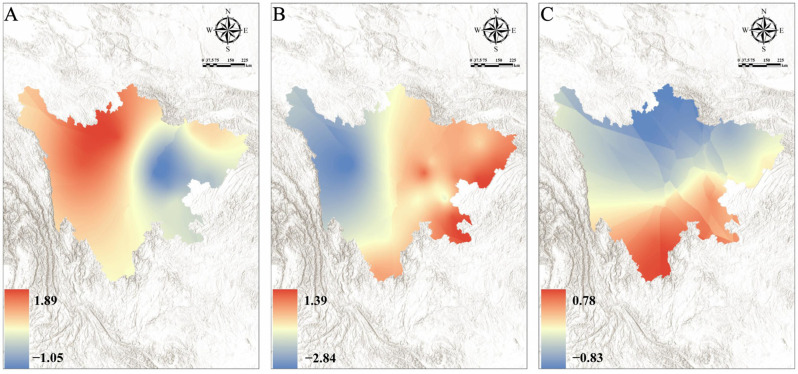
Spatial trend surfaces of M_DH RSRS (**A**), M_N RSRS (**B**), and M_HT RSRS (**C**) at the prefecture level.

Trend surface analysis at the prefecture-level scale (n = 21) showed that M_DH RSRS had a significant northwest-high to southeast-low gradient (first-order trend surface: F = 8.34, *p* = 0.003, R^2^ = 0.46, and LOO-CV R^2^ = 0.38; partial regression coefficient for latitude beta = 0.41, *p* = 0.007; and longitude beta = −0.29, *p* = 0.031) (Figure 10). This gradient was highly consistent with the spatial gradient of CASI. The trend surface for M_N RSRS was also significant (F = 5.87, *p* = 0.011, and R^2^ = 0.38), but it showed an east-high to west-low pattern (longitude beta = 0.44, *p* = 0.009). This suggests spatial coupling between high fertilizer input and nutritional stress responses in hilly and low-mountain areas. For M_HT, the trend surface had a negative coefficient for latitude (beta = −0.33, *p* = 0.044), showing that the molecular heat-stress response became stronger toward southern, lower-latitude regions. The trend surfaces for M_OS and M_CS did not reach significance. At the county scale (n = 183), the second-order trend surface of CASI further improved the goodness of fit (R^2^ = 0.67, LOO-CV R^2^ = 0.63; see Supplementary Table S6 and Figure S4). The pattern formed a concentric zonal structure, with values increasing outward from the Chengdu Plain as a low-value core toward the northwestern plateau and the Daba Mountains. This spatial pattern was highly consistent with the known agroecological zonation of Sichuan Province and provided geographical continuity evidence for the cross-scale coupling.

### 3.5. External Validation Using Independent Field RNA-seq Datasets

In this section, five independent field RNA-seq datasets were used for external validation (GSE97205, GSE166348, GSE124100, GSE142477, and GSE153150; 31 samples in total; stress group, 19; and control group, 12). These datasets were not used in the module definition described in Section 3.2. The validation was arranged in the following way. WGCNA module identification and hub-gene annotation were first carried out with 15 controlled-experiment datasets. After this step, the five field datasets were treated as independent new batches and entered into ComBat-seq correction, using dataset source as the batch factor and stress-intensity grade as the covariate. The corrected data were then projected onto the predefined gene modules, and GSVA scores were compared. This design kept the field validation externally independent and reduced the risk of circular reasoning. For all samples, STAR alignment rates were higher than the 70% quality threshold (range, 73.8–87.3%; mean, 80.2%; Supplementary Table S2). Stress grades were assigned independently according to meteorological records, with severe stress defined as precipitation anomaly < −60% or temperature ≥ 37 °C, moderate stress as −60% ≤ precipitation anomaly ≤ −40% or temperature 35–37 °C, and mild/normal conditions as deviations within ±15%.

#### 3.5.1. Module Level—Three Modules Received Independent Field Support

**Figure 11:**
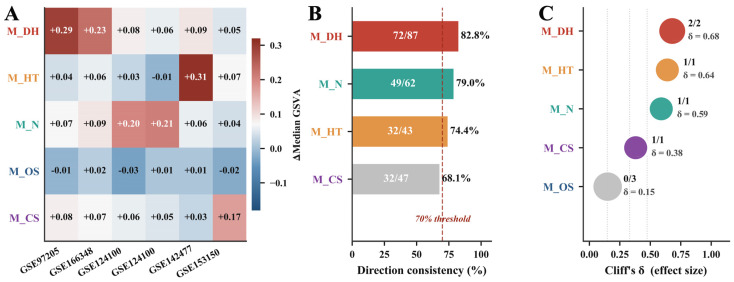
ΔMedian GSVA scores are shown for each module–dataset pair based on five independent field datasets with 31 samples in total; only M_OS did not reach significance (**A**); directional consistency of hub genes (kME > 0.8), with the dashed line marking the 70% validation threshold (**B**); and replication of each module in independent field datasets, including the number of passing datasets (/total number) and Cliff’s δ effect size (**C**).

At the module level, validation was conducted with the Wilcoxon rank-sum test. A module was regarded as passing when BH-adjusted *p* < 0.05 and |ΔMedian GSVA| > 0.1 (Table 6 and Figure 11A). M_DH met this criterion in two independent datasets at the same time, namely, GSE97205 (ΔMedian = +0.287, BH *p* = 0.003) and GSE166348 (+0.231, BH *p* = 0.021), and its Cliff’s δ was 0.68, indicating a large effect. M_N also passed in both tissues of GSE124100, including leaf (+0.198, BH *p* = 0.018) and root (+0.214, BH *p* = 0.011), with Cliff’s δ = 0.59, which was a medium-to-large effect. M_HT was significant in pollen samples from GSE142477 (+0.312, BH *p* = 0.008), and Cliff’s δ was 0.64, also showing a large effect. M_CS passed in GSE153150, which included Sichuan samples (+0.167, BH *p* = 0.034), but this result came from only one dataset, and the effect size was moderate (Cliff’s δ = 0.38). Therefore, M_CS was regarded as a candidate that still needs further testing with more field samples. M_OS did not show significance in any of the five field datasets (BH *p* > 0.05, |ΔMedian| < 0.08) and was therefore given a “neutral conclusion.” This result may be connected with the field stress conditions, which were mainly drought/heat rather than salinity–alkalinity, and it does not change the conclusions obtained from controlled experiments.

#### 3.5.2. Hub-Gene Level—Directional Consistency Supports the Module-Level Results

For the four modules with field support, the directional consistency of differential expression was further checked for their hub genes (kME > 0.8) in the field datasets. The preset rule was that the fold-change directions of at least 70% of hub genes should agree with those observed in the controlled experiments (Figure 11B). The consistency reached 82.8% for M_DH hub genes, with 72 of the 87 genes showing the same direction. It was 79.0% for M_N (49 of 62) and 74.4% for M_HT (32 of 43). These three modules all exceeded the 70% threshold, which supported the module-level results from another angle. For M_CS, the value was 68.1%, slightly lower than the threshold, which matched its module-level status of “preliminary field support.”

#### 3.5.3. Preliminary Regional Applicability of Sichuan-Region Samples

Taken together, the Cliff’s δ effect-size summary across all five modules (Figure 11C) revealed a clear hierarchy of field reproducibility: M_DH (δ = 0.68; 2/2 datasets), M_HT (δ = 0.64; 1/1), and M_N (δ = 0.59; 1/1) all exceeded the conventional large-effect cutoff (δ ≈ 0.47); M_CS (δ = 0.38; 1/1) fell within the medium-effect range, consistent with its “preliminary field support” status; and M_OS (δ = 0.15; 0/3) sat in the negligible-effect range. This effect-size landscape reinforces the qualitative conclusions drawn from Figure 11A,B and provides an integrated view of how strongly each module is recapitulated under realistic field conditions. Notably, within GSE153150—the only field dataset that contained Sichuan-origin samples (n = 3; Supplementary Table S2)—M_CS reached its largest ΔMedian among all field datasets (+0.17), suggesting that the molecular features identified in this study may have preliminary regional applicability for local maize in Sichuan. However, the number of local Sichuan field samples was small (n = 3), so stricter local extrapolation still needs validation with a larger sample set. Even so, this result gives an initial basis for later screening of molecular targets related to stress-resistance breeding and precision agronomic regulation of maize in Sichuan.

## 4. Discussion

At the regional scale, this study built a statistically testable coupling path from macroscopic emergy gradients to maize molecular response modules. The main reason for the robustness of this path is that three inferential methods, which are different but complementary in principle, produced convergent results in the same direction. Sparse canonical correlation analysis can find shared variation directions under small-sample and high-dimensional conditions [20]. Random forests use nonparametric ensemble learning and are relatively insensitive to nonlinear relations and heteroscedasticity [21]. The spatial error model gives conditional regression coefficients after spatial autocorrelation is controlled [22]. These three methods all placed ELR as the main macroscopic variable related to the M_DH module (RF % IncMSE = 28.4; SEM beta = 0.51) and Fn as the main variable related to the M_N module (RF % IncMSE = 31.2; SEM beta = 0.46). This agreement across methods gives a direct response to the possible concern of “spurious correlation” under the small sample size (n = 21). If the coupling had only been caused by sampling fluctuation, or by the assumptions of one specific method, then three approaches based on clearly different principles would not be expected to give results in the same direction and along the same gradient at the same time. It also needs to be stressed that the bias structures of the three methods are not the same. sCCA relies on sparsity assumptions in the X and Y blocks, and the upward bias of small-sample correlation coefficients was controlled by shrinkage correction (r1 shrinks from 0.81 to 0.67–0.71) and leave-one-out cross-validation. Random forest %IncMSE is obtained through the perturbation of out-of-bag samples and does not require distributional assumptions, although it can be affected by split bias among correlated variables. For this reason, it complements the covariance-structure identification of sCCA. In contrast, the spatial error model uses Moran’s I LM diagnostics to choose the appropriate spatial filtering mode (M_DH RSRSI = 0.347, *p* = 0.018), thus avoiding the regression bias that OLS may produce when spatial autocorrelation exists. The RSRS construction based on the GSVA algorithm [36] keeps module activity continuous at the sample level. Therefore, later Lasso [19] and sCCA inferences did not have to depend on binarization or discretization assumptions. ComBat-seq negative-binomial batch correction [32] also reduced the variance explained by batch effects across 15 public datasets from R^2^ = 0.41 to 0.09 and increased the variance explained by stress type from 19.8% to 31.4%. This clear improvement in the signal-to-noise ratio is an important precondition for reliable module identification. It should be stated clearly that the lower bounds of the bootstrap 95% CIs for the RF variable importance of M_OS and M_CS crossed 0. Thus, this study treats them explicitly as non-independent inferences. Such a cautious treatment reflects the use of small-sample statistical robustness principles rather than an attempt to avoid the limitations of the results.

The direction of the coupling relationship has a reasonable biological explanation, and it should not be regarded as only an accidental output of data-driven analysis. ELR describes the relative pressure of nonrenewable emergy compared with local renewable emergy [24]. Counties with high ELR are usually linked with unbalanced chemical fertilizer and irrigation inputs, declining soil quality, and stronger microclimatic evapotranspiration. These conditions are exactly the physical basis that can activate the dehydration-response cascade. The main hub in the M_DH module, ZmDREB2A (kME = 0.91, degree = 47), belongs to the classical ABA-independent DREB2 transcriptional pathway. Its coordinated responses with downstream LEA protein genes and HSP-encoding genes under the combined stresses of dehydration and heat shock form a central molecular framework that has been repeatedly supported in studies of plant stress regulation [11,42]. The synchronous clustering of ZmHSP101 (kME = 0.88), ZmABF2 (0.87), and ZmLEA3 (0.84) inside M_DH also supports the classical view that DREB2, HSF, and bZIP transcription factors are coordinately upregulated under combined drought–heat stress. In M_HT, ZmHSFA2 (kME = 0.92) is the strongest hub, and this is highly consistent with recent work on the ZmHSF20-ZmHSFA2 regulatory loop [12,13,43]. The ZmHSFA2 autoregulatory circuit and the ZmHSF20-ZmHSF4-ZmCesA2 module together form the core transcriptional framework of maize heat response. This study further establishes, at the regional scale, a quantifiable statistical coupling between this experimentally controlled framework and the macroscopic temperature gradient. Fn represents the dependence on anthropogenic-source nitrogen relative to natural-source nitrogen. Its increase directly corresponds to the high-affinity nitrate transport pathway dominated by ZmNRT2.1 in the M_N module [15]. The multi-stress cross-regulation mediated by ZmWRKY33 [14] also supports the interpretability of M_N. In a broader sense, the pattern found here, that is, “compound macroscopic stress driving synchronous responses across multiple modules,” is consistent with the argument of Mittler (2006) [44] and Suzuki et al. (2014) [45] that combined stress induces distinctive transcriptomic features. It also agrees with the view of Zandalinas et al. (2021) [46] on the synthetic effects of multifactorial stress, where even weak, single stresses may cause nonadditive plant damage when they are superimposed. Two topological features further support this interpretation. The internal clustering coefficient of M_DH (0.56) is significantly higher than those of the other four modules, and the four cross-module hubs are located at the intersection of the three major response pathways in the “drought-heat-nitrogen-stress cascade.” These features agree with the maize stress-response network framework indicated by STRING [17] and PlantRegMap [18] annotations. This network structure suggests that the macroscopic emergy structure “penetrates” into cellular-molecular signaling pathways through several nodes with high betweenness, especially the convergence points of ROS and hormone signaling [47]. This mechanistic correspondence is difficult to identify using only climate–yield statistical models [1,2,3].

Beyond confirming the well-characterized cross-module hub genes, the co-expression network also identified a set of functionally uncharacterized genes that ranked highly in terms of data-driven contribution (Section 3.2; Supplementary Table S7). These candidates may be of particular interest for future investigation. Because they co-cluster closely with the conserved hubs of their respective modules, the principle of guilt-by-association suggests that they may perform previously undescribed functions within the corresponding stress and nutrient-response pathways. Accordingly, we regard these genes not as confirmed regulators but as data-nominated, high-priority candidates for subsequent functional characterization and regulatory pathway analysis, including expression validation, mutant or gene-editing experiments, and regulatory network reconstruction. As these assignments are based on computational predictions, experimental validation will be required. Nevertheless, these candidates represent a promising avenue for further elucidating the molecular mechanisms underlying maize stress responses and adaptation.

The conclusions are also supported by dual robustness at the spatial level and the external-data level, which strengthens their extrapolability. The four functional zones identified by PLS-DA (Q^2^ = 0.548), together with the county-level (n = 183) second-order trend surface (R^2^ = 0.67; LOO-CVR^2^ = 0.63), show that M_DH RSRS and CASI form a continuous geographic gradient from high values in the northwest to low values in the southeast. This pattern is therefore not a discrete artifact caused by the 21 sampling sites. The gradient has a clear background. In the main maize-producing areas of Sichuan, the terrain–climate transition from basin plains, to basin hills and low mountains, and then to plateaus and dry-hot valleys provides a natural experimental field for the hypothesis of spatial nesting of multifactorial stress [4,5]. It is also highly consistent with the principle of multifactorial stress accumulation emphasized by Zandalinas et al. (2021) [46]. The two-layer validation using five independent field RNA-seq datasets further indicates that the major conclusions of the framework can be reproduced outside the training data. M_DH passed independent validation in GSE97205 and GSE166348 (Cliff’s delta = 0.68), M_N passed validation in two tissues in GSE124100 (delta = 0.59), and the directional consistency of core genes reached 82.8% for M_DH, 79.0% for M_N, and 74.4% for M_HT. It should be noted that the “model first, then projection” validation procedure ensured strictly that the field datasets were not used in WGCNA module definition. At the same time, treating them as new batches recalibrated by ComBat-seq [32] helped to avoid circular reasoning. This step has been relatively insufficient in earlier regional integration studies. The significant upregulation of M_HT in the pollen-specific dataset GSE142477 (Cliff’s delta = 0.64) is in line with reproductive-stage heat sensitivity mediated by ZmHSFA2 (Ruan et al., 2024) [43]. In GSE153150, the Sichuan regional samples (n = 3) showed higher GSVA scores in the M_DH module than non-Sichuan samples (DeltaMedian higher by 0.09–0.14). This observation is geographically consistent with the “high-ELR areas in plateau prefectures and along the periphery of the basin” reported by Yang et al. (2025) [48] in the emergy evaluation of maize ecosystems across 21 prefecture-level units in Sichuan Province. It therefore provides independent emergy-accounting support for the regional specificity of molecular module activity.

The application boundaries of this study should still be defined carefully. M_OS did not receive significant support in any of the five field datasets. Its signal in controlled experiments may have appeared because the salinization simulation was stronger than actual field conditions in Sichuan. This agrees with the caution raised by Suzuki et al. (2014) [45] that controlled environments may enlarge the signal of a single stress. Therefore, this study classifies M_OS as “conclusion-neutral” rather than as one of the core conclusions. The number of field samples containing the Sichuan region is only three, so they can only provide preliminary evidence of regional applicability for local extrapolation. This evidence still needs to be confirmed by larger local field omics cohorts. The current coverage of emergy parameters and climatic gradients [10] also means that the weighting scheme must be recalibrated before the framework is extended to the North China Plain or the black-soil region of Northeast China. Under these limitations, the methodological contributions of this study can be summarized in at least three aspects. First, it systematically links emergy accounting [8,9,24,48] with the maize stress transcriptome at the regional scale and establishes a continuous inferential route from emergy flows to regulatory networks. Second, it uses the same-direction convergence of sCCA, random forest, and the spatial error model [19,20,21,22] as a robustness threshold for small-sample cross-scale inference. This provides a methodological template that can be transferred to similar regional integration studies. Third, the external validation paradigm of “model first, then projection + ComBat-seq batch correction + multi-dataset Cliff’s delta validation” can be applied to other cross-database integration studies in crop science [32,43,47]. In practical terms, the findings can provide a quantifiable basis for precision variety regionalization and differentiated nitrogen-fertilizer management through CASI zoning in the main maize-producing areas of Sichuan [48]. At the same time, this study offers a reusable paradigm for moving climate–yield risk assessment toward the level of molecular mechanisms. This responds to the current methodological need in the academic community to study crop adaptability under normalized scenarios of multifactorial combined stress [4,5,46].

Although the present analysis was anchored in the maize-producing system of Sichuan, the value of the proposed framework lies less in its region-specific numerical outputs than in its transferable, modular architecture. The five-step closed-loop design—construction of a macro-scale composite stress index, data-driven identification of molecular response modules, multi-method consistency inference for cross-scale coupling, spatial-continuity testing, and independent field validation—is method-agnostic: each component can be re-parameterized or substituted without altering the overall logic. The molecular anchors were deliberately derived from a broad-provenance public corpus spanning diverse genetic backgrounds and experimental settings rather than from region-specific samples so that the five modules, the 270 core genes, and the four cross-module hubs (ZmDREB2A, ZmHSFA2, ZmWRKY33, and ZmNRT2.1, all conserved master regulators of abiotic-stress signaling) are expected to function as generalizable molecular references across genotypes. Likewise, CASI is a reconstructable index rather than a fixed scoring rule: in a new region, the same VIF-based screening and principal-component weighting can be re-applied to the locally available emergy, climate, soil, and terrain variables. The “model-first, projection-later” validation design and the three-method (sCCA, random forest, and spatial error model) triangulation are portable safeguards against circular reasoning and small-sample false positives and can be reused in any cross-scale ecological–molecular study. Together, these features indicate that the framework is, in principle, transferable to other cereal crops and to other major maize ecological regions, such as the North China Plain and the black-soil belt of Northeast China.

A further consideration concerns genotype: the cultivars grown in the study area differ from those represented in the public datasets used for module construction, and genotype-by-environment (G×E) interactions cause cultivars to differ in their absolute tolerance to environmental stress. Our design and inference were scoped to remain valid under this condition. The molecular anchors were intentionally derived from a broad-provenance corpus spanning diverse genetic backgrounds rather than from any single cultivar (Section 2.2) so that the five modules and their hubs—ZmDREB2A, ZmHSFA2, ZmWRKY33, and ZmNRT2.1, conserved and functionally characterized regulators of abiotic-stress and nitrogen responses—capture the core, genotype-shared regulatory machinery engaged by each stress type rather than cultivar-specific quantitative tolerance. Our conclusions are correspondingly framed as reproducible, direction-consistent associations between macro-scale stress axes and the activation of these conserved modules, not as cultivar-specific tolerance rankings. Importantly, robustness to genotype was tested empirically: the core modules (M_DH, M_HT, and M_N) were reproduced as large-effect signals across five independent field datasets originating from markedly different genetic backgrounds and growing environments, and the Sichuan-origin samples (GSE153150) offered preliminary, locally matched corroboration—indicating that the identified module–stress relationships are not artifacts of the particular cultivars used for module construction. Accordingly, the practical value of the framework lies in stress-intensity zoning and in the prioritization of conserved molecular targets and management foci (for example, differentiated nitrogen management and drought- and heat-oriented varietal screening) for each zone rather than in prescriptive, cultivar-specific recommendations. Translating these zone- and module-level priorities into deployable agronomic and breeding decisions for specific local cultivars will require dedicated G×E validation using Sichuan-grown germplasm and larger local omics cohorts, which we identify as the essential next step.

Several limitations nonetheless define the application boundaries of this study and should temper interpretation. First, the macro-scale evidence chain rests on only 21 prefecture-level units; despite triangulation, bootstrap confidence intervals, and multiple-testing correction, the inference operates under a small-sample, high-dimensionality regime, and the coupling relationships are best read as reproducible statistical associations rather than as mechanistic causation, which will require dedicated functional or controlled-gradient experiments to establish. Second, the CASI weighting scheme is calibrated to the variable structure and the southeast-low/northwest-high gradient of Sichuan and must be recalibrated before transfer to ecologically distinct regions. Third, the molecular response phenotype was obtained by projecting stress-type module scores onto prefecture units through a mapping that is constrained to be independent of the CASI predictors; because the public RNA-seq corpus carries no field geolocation, this projection remains an approximation. Fourth, the field-level confirmation of regional applicability relied on only three Sichuan-origin samples (GSE153150), so this finding is reported as preliminary and warrants verification with larger local omics cohorts. Fifth, transferability is module-dependent: the osmotic/salt module (M_OS) was not reproduced in any field dataset, most plausibly because controlled salinity simulations exceeded the salinity stress actually encountered in Sichuan fields, indicating that per-module portability depends on the correspondence between the field stress spectrum and the stress type a module represents. Finally, the framework integrates climatic normals, emergy/management statistics, and transcriptomes obtained in different periods as a static, cross-sectional snapshot; interannual variability, developmental-stage effects, and temporal dynamics are therefore not captured and remain an important direction for future longitudinal extensions.

## 5. Conclusions

This study established a reproducible and auditable cross-scale framework that links regional, emergy-based agroecological stress to maize transcriptomic responses under a small-sample constraint (n = 21). The composite abiotic stress index (CASI) exhibited a stable, spatially interpolable terrain-driven gradient across Sichuan, whereas the maize stress transcriptome resolved into five modules and 270 core genes organized around four cross-module hubs (ZmDREB2A, ZmHSFA2, ZmWRKY33, and ZmNRT2.1). Three methodologically independent inference approaches—sparse canonical correlation analysis, random forest, and the spatial error model—converged to identify the ELR–M_DH and Fn–M_N couplings as reproducible statistical associations, which were further corroborated by five independent field RNA-seq datasets. By integrating emergy accounting, spatial statistics, co-expression networks, and robust small-sample inference, the framework offers an operational basis for precision varietal zoning and differentiated nitrogen management and is transferable—after region-specific recalibration—to other major maize ecological regions of China.

## Figures and Tables

**Table 1:** PCA results for CASI and the weights of the nine variables.

Principal Component/Variable	Eigenvalue/Weight	Explained Variance/Bootstrap 95% CI	Main Content/Interpretation
PC1	4.25	47.3%	ELR, Fn, SMD (inverted)
PC2	1.99	22.1%	Mean annual temperature, SPEI, annual precipitation
PC3	1.29	14.3%	Soil pH, soil organic matter
ELR	0.189	0.152–0.221	Largest weight (mainly chemical-nutritional stress)
Fn	0.171	0.138–0.198	Second largest weight (nonrenewable anthropogenic auxiliary emergy)
Mean annual temperature	0.138	0.108–0.164	Mainly climatic stress
SPEI (3-month scale)	0.124	0.094–0.152	Drought stress
SMD (inverted)	0.112	0.081–0.141	Terrain-related physical stress
NEYR (inverted)	0.108	0.076–0.137	Chronic stress at the system level
Soil pH	0.097	0.065–0.126	Soil chemistry
Annual precipitation	0.090	0.061–0.118	Climatic stress
Soil organic matter	0.083	0.058–0.107	Soil physical-chemical properties

**Table 2:** One-way analysis of variance and Tukey HSD post hoc comparison of CASI among the four terrain divisions.

Terrain Division	n	CASI Mean ± SD	vs. Basin Plain (*p*)	vs. Basin Hills (*p*)
Basin plain	5	−0.83 ± 0.31	-	0.038
Basin hills	8	0.12 ± 0.44	0.038	-
Low-mountain areas	5	0.76 ± 0.38	<0.001	0.012
Plateau/high mountains/dry-hot valleys	3	1.94 ± 0.41	<0.001	<0.001

**Table 3:** Main characteristics of the five stress-responsive gene modules.

Module	Gene Number	Major Stress Type	rho (Module Stress)	Adjusted *p*	Core Gene Number (kME > 0.8 and |GS| > 0.5)
M_DH	1243	Combined drought/heat stress (including osmotic stress)	0.78	<0.001	87
M_HT	634	Reproductive-stage heat stress (pollen/silk)	0.72	<0.001	43
M_N	891	Low-nitrogen/low-phosphorus nutrient stress	0.69	<0.001	62
M_OS	478	Osmotic/saline–alkali stress	0.58	0.007	31
M_CS	712	Chronic combined stress (superimposition of multiple stresses)	0.54	0.021	47

Note: rho refers to Spearman’s rank correlation coefficient between the module eigengene (ME) and the corresponding stress phenotype variable; *p* values were corrected by Bonferroni.

**Table 4:** Random forest variable importance and concordance with sCCA.

Module	RF First Variable (%IncMSE; 95% CI)	RF Second Variable	RF Third Variable	Number of Stable Variables	sCCA/RF Concordance
M_DH	ELR (28.4; 14.2–43.1)	CASI (24.7)	Mean annual temperature (19.3)	5/9	Top three concordant
M_HT	Mean annual temperature (35.7; 19.2–52.1)	SMD (22.1)	SPEI (13.8)	3/9	Top three concordant
M_N	Fn (31.2; 12.8–49.6)	ELR (23.8)	Soil pH (18.4)	4/9	Top three concordant
M_OS	Annual precipitation (9.3; −2.1–21.4)	SPEI (8.6)	CASI (7.2)	0/9	CI lower bound < 0; unstable
M_CS	CASI (11.2; −1.3–24.8)	ELR (9.8)	Fn (8.1)	0/9	CI lower bound < 0; unstable

**Table 5:** Summary of spatial regression (SEM/OLS) estimates.

Model	Response Variable	Key Predictor	Beta (Standardized)	Beta 95% CI (Bootstrap)	*p* Value	R^2^/pseudo-R^2^
SEM	M_DH RSRS	ELR	0.51	0.32–0.71	0.008	pseudo-R^2^ = 0.58
SEM	M_DH RSRS	Mean annual temperature	0.38	0.18–0.58	0.024	Same as above
SEM	M_N RSRS	Fn	0.46	0.26–0.66	0.012	pseudo-R^2^ = 0.47
SEM	M_N RSRS	Soil pH	0.33	0.12–0.54	0.039	Same as above
OLS	M_HT RSRS	Mean annual temperature	0.43	0.19–0.67	0.031	R^2^ = 0.28
OLS	M_OS RSRS	Annual precipitation	0.22	−0.08–0.52	0.142	R^2^ = 0.18
OLS	M_CS RSRS	CASI	0.19	−0.11–0.49	0.198	R^2^ = 0.21

Note: SEM was applied to response variables with significant Moran’s I (M_DH and M_N), and OLS was applied to the remaining variables. The 95% CIs of beta were calculated from bootstrap n = 1000. pseudo-R^2^ was calculated by the Nagelkerke method.

**Table 6:** Cross-validation and effect-size assessment of five stress-response modules in field RNA-seq datasets.

Module	Field Dataset	ΔMedian GSVA	BH-Adjusted *p*	Datasets Passing	Cliff’s δ	Field Conclusion
M_DH	GSE97205, GSE166348	+0.287/+0.231	0.003/0.021	2/2	0.68	Two-dataset support
M_HT	GSE142477 (pollen)	+0.312	0.008	1/1	0.64	Single-dataset support
M_N	GSE124100 (leaf/root)	+0.198/+0.214	0.018/0.011	1/1 (two tissues)	0.59	Two-tissue support in one dataset
M_CS	GSE153150 ★	+0.167	0.034	1/1	0.38	Preliminary support (candidate)
M_OS	GSE97205, etc.	<0.08 (all nonsignificant)	>0.05	0/3	0.15	No independent replication

Note: Passing was defined by |ΔMedian GSVA| > 0.1 and BH-adjusted *p* < 0.05. Passing in ≥2 independent datasets was considered field support, while passing in 1 dataset was considered preliminary field support. Cliff’s δ was interpreted according to Cliff (1993) [41]: |δ| < 0.147, negligible; 0.147–0.33, small; 0.33–0.474, medium; and ≥0.474, large. ★ GSE153150 contains samples from the Sichuan region.

## Data Availability

The original contributions presented in this study are included in the article. Further inquiries can be directed to the corresponding authors.

## Supplementary Materials

The following supporting information can be downloaded at: https://www.mdpi.com/article/10.3390/plants15131977/s1. Methods S1: Correspondence between transcriptomic data sources and regional eco-geographical stress axes; Methods S2: Comparison of ComBat-seq batch-correction strategies and evaluation of overcorrection risk; Methods S3: WGCNA network construction parameters, soft-threshold scanning, and module identification; Methods S4: Statistical robustness assessment standard and full statistical reporting criteria for small-sample (n = 21) cross-scale analysis; Methods S5: Full procedure for variance inflation factor (VIF)-based screening of multicollinearity among candidate variables; Methods S6: Robustness assessment procedure for CASI weights based on bootstrap and leave-one-out (LOO) methods; Table S1: Basic information for 15 public RNA-seq datasets; Table S1-b: Correspondence between each module-construction dataset, its stress type, and the regional eco-geographical stress axis quantified by CASI; Table S2: Sample information for five independent field RNA-seq datasets; Table S3: Stepwise VIF screening of multicollinearity for 14 candidate variables; Table S4: Robustness assessment of CASI weights using bootstrap (n = 1000) and leave-one-out evaluation; Table S5: Core gene list of 270 genes (kME > 0.8 and |GS| > 0.5); Table S5-b: Shrinkage-corrected and LOO-CV statistics for the first two pairs of canonical variables in sCCA; Table S6: Detailed statistics for the county-scale (n = 183) second-order CASI trend surface; Table S7: Functionally uncharacterized, high-contribution candidate genes nominated by the cross-scale framework; Figure S1: WGCNA soft-threshold β scanning curve; Figure S2: Bootstrap distribution histogram of CASI weights; Figure S3: PCoA of RNA-seq expression profiles before and after batch effect correction; Figure S4: County-scale CASI trend surface. Refs. [32,49,50] are cited in the Supplementary Materials file.
