# Supplementary material for: Co-Expression Modules and Core Regulatory Factors Linked to Maize Abiotic Stress Resistance Under the Compound Agroecological Stress Index in Southwest China
Source: Plants (Basel). 2026 Jun 26;15(13):1977. doi: 10.3390/plants15131977 (PMC13363898; doi:10.3390/plants15131977)
Supplement: Supplementary file 1 [file plants-15-01977-s001.zip › plants-4334914-supplementary.pdf]

Supplementary Materials:

Part I Supplementary Methods

S1 Correspondence between transcriptomic data sources and regional eco-geographical stress axes

The transcriptomic data used in this study were organized into two tiers that play deliberately different roles, and eco-geographical correspondence is required only at the cross-scale coupling and external-validation tiers, not at the module-construction tier. The 15 module-construction datasets (286 samples; Supplementary Table S1) were selected on the basis of stress type (drought/water deficit, heat, low nitrogen/low phosphorus, and combined stress) and uniform technical quality criteria (Illumina NovaSeq 6000 or HiSeq series; PE150; sample size per group greater than or equal to 3; sequencing depth greater than or equal to 20 M reads/sample; publicly retrievable fastq). Geographic origin was not an inclusion criterion, because these datasets are controlled, stress-type-resolved experiments whose purpose is to define separable co-expression modules and hub genes that function as generalizable molecular anchors. Broad provenance across diverse genetic backgrounds therefore strengthens, rather than weakens, the transferability of the resulting modules, and a field eco-geographical origin is not the pertinent attribute for these controlled datasets.

The alignment between these molecular anchors and the eco-geographical factors of the study region is established through stress-axis correspondence and is then tested statistically. The four stress categories represented in the module-construction data map directly onto the eco-geographical stress dimensions quantified by the nine CASI variables in Sichuan: drought/water deficit and heat correspond to the climatic-hydric and climatic-thermal axes (SPEI-3, annual precipitation, mean annual temperature); low nitrogen/low phosphorus and combined stress correspond to the chemical-nutritional and emergy axis (ELR, Fn, NEYR) conditioned by soil chemistry (soil pH, soil organic matter); and these are further modulated by the terrain physical-stress axis (SMD). This dataset-to-axis mapping is given explicitly in Supplementary Table S1-b. The conceptual correspondence is subsequently subjected to formal statistical testing through the cross-scale coupling analysis in the main text (Section 3.3), in which sparse canonical correlation analysis, random forest, and the spatial error model converge on the ELR to M_DH and Fn to M_N relationships.

Empirical field and spatial eco-geographical correspondence is provided by the five independent validation datasets (Supplementary Table S2), which, unlike the module-construction data, are geographically explicit. These datasets span representative maize agro-ecological regions (North and East China, the Eastern European Plain, the U.S. Corn Belt, New South Wales in Australia, and China including Sichuan), do not overlap with the module-construction data, and enter the analysis only through the model-first, projection-later workflow with ComBat-seq re-batching, so that they do not participate in module definition. Critically, GSE153150 contains Sichuan-origin samples (n = 3) drawn from the hill to low-mountain transition zone of the Sichuan Basin (Zigong, Nanchong, and Guangyuan), in which the M_DH module reached its largest median GSVA difference among all field datasets. This constitutes direct, although preliminary, evidence that modules built from geographically diverse controlled data recapitulate in the target eco-geographical setting. The small number of Sichuan-origin samples (n = 3) is stated as a limitation in the main text, and local extrapolation should be confirmed with larger regional field cohorts.

S2 Comparison of ComBat-seq batch-correction strategies and evaluation of overcorrection risk

This study combined 15 public RNA-seq datasets (Supplementary Table S3), and dataset origin was the main known batch factor. To reduce batch effects while preserving as much as possible the biological signals related to stress type, ComBat-seq [2] was used for empirical Bayes batch correction of the count matrix.

S2.1 Main correction scheme

"Dataset origin" was defined as the batch factor, while "stress type" (drought/water deficit, heat stress, low nitrogen/low phosphorus, and combined stress) was defined as the protected covariate, namely the covariate of interest. The parameter settings were group = stress type, covar_mod = NULL, and shrink = TRUE. For low-expression filtering, the criterion was CPM > 1 in at least three samples for each gene. After correction, PERMANOVA with 999 permutations showed that the variance explained by dataset origin decreased from $R^2 = 0.41$ ($p < 0.001$) to 0.09 ($p = 0.112$), while the variance explained by stress type increased from 19.8% to 31.4% ($p < 0.001$). Therefore, the batch effect was not statistically significant after correction, whereas the stress signal became clearer and stronger. This pattern was consistent with the expected behavior of an appropriate correction (see Supplementary Figure S3).

S2.2 Negative-control sensitivity analysis

To rule out the possibility of "overcorrection," meaning that true biological signals were wrongly removed as batch effects, three negative controls were used. First, a set of housekeeping genes weakly related to stress was selected, including 120 genes such as GAPDH, ACTIN, EF1α, and 18S rRNA, and their expression variance was expected to remain stable before and after correction. Second, ComBat-seq was rerun after the batch labels were randomly permuted, and the variance explained by stress was not expected to increase clearly. Third, an intentionally wrong correction was performed by treating stress type as the batch factor, and the expected result was a collapse of the variance explained by stress. The results showed that the variance of the housekeeping gene set changed by only 2.3% before and after correction. Under randomized batch labels, the variance explained by stress was 18.6%, which was almost the same as the value before correction. After the deliberately wrong correction, the variance explained by stress decreased to 4.1%. All three negative controls were passed, showing that the main correction scheme did not introduce overcorrection.

S2.3 Application of the corrected expression matrix

The corrected expression matrix was then used in the later analysis workflow. First, after low-expression filtering, 23,847 genes were retained. Second, outlier samples were identified using dynamic tree cutting based on between-sample correlations, and seven outlier samples were removed. Third, the remaining 279 samples were used to construct the WGCNA network.

S3 WGCNA network construction parameters, soft-threshold scanning, and module identification

S3.1 Soft-threshold β scanning

The scale-free topology fitting index $R^2 \geq 0.85$ was used as the main criterion, and the decline rate of mean connectivity was also followed. β was scanned from 1 to 20, and the R^2 and mean connectivity of the whole network were calculated at each β value (Supplementary Figure S1). At $\beta = 13$, R^2 first reached 0.843. At $\beta = 14$, $R^2 = 0.871$, which was the first value above the 0.85 threshold, while mean connectivity decreased to 23.4, suggesting a moderate level of network

sparsity. Increasing β further to 15–20 produced less than 2% additional R^2 gain, but it made the network excessively sparse. Therefore, β = 14 was finally selected.

S3.2 Adjacency matrix and topological overlap matrix

A weighted adjacency matrix based on Pearson correlation coefficients was defined as $a_{ij} = |cor_{ij}|^\beta$. This matrix was then converted into a topological overlap matrix (TOM), and 1 - TOM was used as the gene-to-gene distance metric. Hierarchical clustering of the distance matrix was conducted with the average linkage method.

S3.3 Dynamic tree cutting and module merging

The parameters for dynamic tree cutting were deepSplit = 2, minModuleSize = 50, pamRespectsDendro = FALSE, and mergeCutHeight = 0.25. This means that modules were merged when the module eigengene correlation was greater than 0.75. The initial cutting step generated 22 modules, and these were reduced to 18 modules after merging. Five modules were significantly related to stress phenotypes ($|\varrho| > 0.5$ and Bonferroni-corrected $p < 0.05$), including M_DH, M_HT, M_N, M_OS, and M_CS.

S3.4 Hub gene screening

The hub gene screening used two thresholds, kME > 0.8 and |GS| > 0.5. Here, kME describes the connection strength between a gene and the eigengene of its assigned module, while GS (gene significance) describes the correlation strength between a gene and the stress phenotype. Across the five modules, 270 hub genes were identified in total, and the complete list is provided in Supplementary Table S2.

S3.5 Functional enrichment and network topology

GO and KEGG enrichment analyses were performed with clusterProfiler using BH-corrected FDR < 0.05. Interactions from STRING (confidence ≥ 0.7), PlantTFDB, and published literature were further combined to build an integrated regulatory network for stress responses. The global network included 270 nodes and 1,834 interaction edges. Its average node degree was 13.6, average clustering coefficient was 0.41, average shortest path length was 3.2, and power-law exponent was $\gamma = 2.34$ ($R^2 = 0.89$), showing typical scale-free characteristics. Based on betweenness centrality, four cross-module backbone hubs were identified: ZmDREB2A, ZmHSFA2, ZmWRKY33, and ZmNRT2.1.

S4 Statistical robustness assessment standard and full statistical reporting criteria for small-sample (n = 21) cross-scale analysis

Because the cross-scale integration was limited by the small sample size of n = 21, this study followed a triangulated-evidence principle when reporting any key inference. The evidence included: p values after multiple-testing correction; Bootstrap 95% confidence intervals for effect sizes; and agreement across at least two independent statistical methods. When all three conditions were met at the same time, the evidence was regarded as relatively strong. If any condition was not met, the relationship was defined as a candidate relationship and was not included in the main conclusions.

S4.1 Multiple-testing correction

Module-phenotype correlations were corrected by Bonferroni correction, with the correction factor equal to 18 modules × 7 phenotypes = 126. Wilcoxon tests for GSVA scores were corrected with the Benjamini-Hochberg procedure to control FDR. The p values in canonical correlation analysis were corrected by Bonferroni correction, with a correction factor of two

pairs of canonical variables. Only results that remained significant after correction entered the next evaluation criterion.

S4.2 Effect sizes and Bootstrap confidence intervals

For relationships involving continuous variables, Cohen's d for GSVA differential scores or the standardized regression coefficient β was used. For rank-correlation conclusions, Spearman ϱ was used, and for group differences, Cliff's δ was used. The interpretation thresholds for Cliff's δ were $|\delta| < 0.147$, negligible; 0.147–0.33, small; 0.33–0.474, medium; and ≥ 0.474, large [3]. All effect sizes were reported together with 95% confidence intervals obtained from Bootstrap resampling with n = 500–1,000 iterations. An effect was considered robust only when the lower bound of the CI was greater than 0, or when the CI did not cross 0.

S4.3 Consistency among independent methods

Main conclusions on cross-scale coupling required support in the same direction from at least two independent statistical methods. For example, the loadings of the first pair of canonical variables in sCCA needed to be consistent with the top three variables ranked by random forest %IncMSE. In this study, the top-three ranking of ELR/CASI/annual mean temperature for M_DH was fully consistent. Another example was agreement between significant driving variables in SEM and stable loadings in sCCA. For modules without sufficient Bootstrap robustness, such as M_OS and M_CS, their variable-importance rankings were not used for independent inference and were reported only as candidate hypotheses.

S4.4 Bias correction for small-sample canonical correlation coefficients

Canonical correlation coefficients from small-sample sCCA have a known tendency to be inflated upward. For each pair of canonical variables, this study reported three types of statistics at the same time: p values before and after permutation-test correction; shrinkage-corrected r based on diagonal shrinkage for bias correction; and leave-one-out cross-validated r (LOO-CV r). A relationship was included in the main conclusions only when all three statistics pointed to a stable relationship in the same direction. For example, CV1 r_1 = 0.81, shrinkage r = 0.67, and LOO-CV r = 0.71 indicated a robust relationship, while CV2 r_2 = 0.67 with corrected p = 0.082 was treated as a candidate relationship.

S5 Full procedure for variance inflation factor (VIF)-based screening of multicollinearity among candidate variables

To keep the composite abiotic stress intensity index (CASI) statistically identifiable under the small-sample condition of n = 21, multicollinearity diagnostics were carried out for the 14 candidate agroecological indicators. Before PCA, all indicators were standardized by Z-score transformation. For indicators that needed direction reversal (NEYR and SMD), negative values were first generated and then standardized, so that all indicators followed the same direction, where larger values represented stronger stress.

S5.1 VIF threshold setting and its theoretical basis

A stepwise threshold rule was applied in the diagnosis. VIF < 5 was considered to indicate acceptable multicollinearity. Values of 5 ≤ VIF < 10 were treated as borderline and were judged together with domain knowledge and the stability of the indicator contribution to CASI weights. VIF ≥ 10 was treated as serious multicollinearity and the variable was excluded directly. Referring to the suggestions of Hair et al. (2014)[1] for multi-indicator social-ecological composite assessment with small samples, and considering that the sample

size in this study was only n = 21, the decision boundaries used here were slightly stricter than conventional criteria.

S5.2 Order for variable deletion and rules for substitution

An iterative screening procedure was adopted. In each round, the variable with the highest VIF was deleted, after which VIFs were recalculated, instead of deleting several variables at one time. When two variables both showed VIF ≥ 10 and also had close conceptual connections, the variable more directly related to the main line of "chemical-nutritional stress" was kept. When their functions largely overlapped (for example, Fn and the inverse of EYR), the variable with higher information content and wider support in previous literature was retained. Five iterative rounds were completed in total. This process removed five variables, namely total radiation, carbon-nitrogen ratio, population density, irrigation-area ratio, and total fertilizer application in kg/ha, and finally kept nine variables for PCA.

S5.3 Sensitivity analysis

To examine whether the VIF screening scheme was robust, CASI was reconstructed and rank correlations were calculated under three alternative schemes: retaining 12 variables, with only variables having VIF > 15 removed; retaining only the seven most core variables with VIF < 3; and using the condition number rather than VIF for the diagnosis. The Spearman correlation matrix showed that the CASI rank correlations between these three schemes and the baseline scheme were 0.91, 0.88, and 0.93, respectively. These values were all within a highly consistent range ($\varrho > 0.85$), which supports the robustness of the baseline scheme. The full records of VIF iteration and the sensitivity matrix are shown in Supplementary Table S4.

S6 Robustness assessment procedure for CASI weights based on Bootstrap and leave-one-out (LOO) methods

Under the small-sample condition of n = 21, PCA loadings may be influenced by single observations. Therefore, two complementary strategies were used to evaluate whether the weighting scheme was statistically robust.

S6.1 Bootstrap resampling (n = 1,000)

Taking the n = 21 prefecture-level units as the population, resampling with replacement was performed to generate 1,000 pseudo-samples, and each pseudo-sample still contained 21 observations. For each pseudo-sample, PCA and weight normalization were repeated, producing 1,000 sets of weights for the nine variables. The 2.5% and 97.5% quantiles of each variable weight were then used as the Bootstrap 95% confidence interval. For all nine variables, the lower bounds of the 95%CIs were above 0 (see Supplementary Table S5 and Supplementary Figure S2), indicating that the weight directions remained stable during resampling.

S6.2 Leave-one-out (LOO)

Each of the 21 prefecture-level units was removed in turn, and CASI construction and ranking were repeated with the remaining 20 samples. This produced 21 LOO-CASI ranking sequences. The Spearman rank correlation coefficient was calculated between each LOO ranking and the original CASI ranking. In the 21 LOO iterations, the mean ϱ was 0.94, the range was 0.89–0.97, and the standard deviation was 0.020. Removing any single sample did not cause a collapse of the ranking results, since all 21 ϱ values were greater than 0.85. This further supports the robustness of the ranking conclusions.

S6.3 Complementary interpretation of Bootstrap and LOO

Bootstrap mainly reflects the parameter uncertainty in weight estimation, especially the sensitivity to numerical loadings. LOO mainly reflects the stability of the sample structure, especially the sensitivity of the ranking results. In this study, the two methods both met the two criteria of "95%CI lower bound > 0" and "$\varrho > 0.85$." Thus, they provided double support for the reliability of CASI as a macro-scale stress indicator. The reporting standard is described in the Methods box on small-sample statistical robustness assessment in the main text, and the complete numerical results are provided in Supplementary Table S5.

Part II Supplementary tables

Supplementary Table S1. Basic information for 15 public RNA-seq datasets.

GEO accession	Year	Sample size	Tissue	Stress type	Maize variety/inbred line
GSE68785	2015	24	Leaf	D	B73 x Mo17 RIL
GSE72302	2015	18	Leaf	H	B73, Mo17
GSE95678	2017	16	Root system	N	B73
GSE104036	2017	20	Leaf	D+H	B73
GSE110391	2018	24	Anther/pollen	H	ZD958
GSE120953	2018	18	Root system	N	B73, Mo17
GSE134070	2019	24	Leaf	D	ZD958, XY335
GSE142889	2020	18	Leaf	C	B73
GSE151878	2020	20	Root system	D+N	B73
GSE158761	2020	16	Leaf	OS	B73
GSE166017	2021	18	Anther	H	ZD958
GSE172203	2021	22	Leaf/root	C	JH series (x4)
GSE179552	2021	14	Leaf	D	tropical inbred lines x3
GSE195234	2022	18	Root system	N+P	B73
GSE210487	2022	16	Leaf	H+D	B73, ZD958

Note: The datasets were collected from NCBI GEO (Gene Expression Omnibus) and MaizeGDB. The whole sample set included 286 samples; 279 samples passed quality control and were used in WGCNA, whereas 7 samples were treated as outliers. The stress-type abbreviations are as follows: D = drought/water deficit (Drought), H = heat stress (Heat), N = low-nitrogen/low-phosphorus nutrient stress (Nutrient), and C = combined stress (Combined). All libraries were sequenced on Illumina

NovaSeq 6000 or HiSeq series platforms with PE150 configuration. Compound codes (e.g., D+H, D+N, N+P) denote co-applied stresses, and OS = osmotic/saline-alkaline stress. Datasets were selected on the basis of stress type and uniform technical quality criteria; geographic origin was not an inclusion criterion (see Supplementary Methods S1). The mapping of each dataset to the eco-geographical stress axes quantified by CASI is given in Supplementary Table S1-b, and the field/spatial eco-geographical correspondence, including Sichuan-origin samples, is documented in Supplementary Table S2.

Supplementary Table S1-b. Correspondence between each module-construction dataset, its stress type, and the regional eco-geographical stress axis quantified by CASI.

GEO accession	Stress type	Corresponding CASI eco-geographical stress axis (key variables)	Represented eco-geographical stressor in the Sichuan study region
GSE68785	D	Climatic-hydric axis (SPEI-3, annual precipitation; mean annual temperature)	Growing-season water deficit across the basin-hill and low-mountain gradient
GSE72302	H	Climatic-thermal axis (mean annual temperature)	High-temperature load in low-altitude basin and dry-hot valley areas
GSE95678	N	Chemical-nutritional / emergy axis (Fn, ELR; soil pH)	Nitrogen-input imbalance under high anthropogenic emergy
GSE104036	D+H	Coupled climatic axis (mean annual temperature x SPEI-3 / precipitation)	Compound drought-heat of the dry-hot valley and low-mountain belt (primary M_DH analogue)
GSE110391	H	Climatic-thermal axis (mean annual temperature)	Reproductive-stage heat in low-latitude / low-altitude zones
GSE120953	N	Chemical-nutritional / emergy axis (Fn, ELR; soil pH)	Nutrient stress under intensive fertilization
GSE134070	D	Climatic-hydric axis (SPEI-3, annual precipitation)	Growing-season drought across the basin-mountain gradient
GSE142889	C	Integrated / chronic-compound axis (ELR, NEYR; climate	Chronic multi-source stress accumulation (M_CS analogue)

GEO accession	Stress type	Corresponding CASI eco-geographical stress axis (key variables)	Represented eco-geographical stressor in the Sichuan study region
		variables)	
GSE151878	D+N	Climatic-hydric x chemical-nutritional axes (SPEI-3 / precipitation x Fn, ELR)	Concurrent water and nitrogen limitation
GSE158761	OS	Osmotic / soil axis (soil pH; salinity not directly represented in CASI)	Osmotic / saline-alkaline stress (limited regional analogue; consistent with the neutral field result for M_OS)
GSE166017	H	Climatic-thermal axis (mean annual temperature)	Reproductive-stage heat
GSE172203	C	Integrated / chronic-compound axis (ELR, NEYR; climate variables)	Chronic compound stress (M_CS analogue)
GSE179552	D	Climatic-hydric axis (SPEI-3, annual precipitation)	Growing-season drought
GSE195234	N+P	Chemical-nutritional / emergy axis (Fn, ELR; soil pH)	Nitrogen and phosphorus input imbalance under high anthropogenic emergy
GSE210487	H+D	Coupled climatic axis (mean annual temperature x SPEI-3)	Compound heat-drought (M_DH analogue)

Note: Geographic origin was not an inclusion criterion for the module-construction datasets (see Supplementary Methods S1). These are controlled, stress-type-resolved transcriptomic experiments selected to characterize separable molecular responses, and they serve as generalizable molecular anchors rather than as region-specific field samples. The correspondence shown here is therefore conceptual and stress-axis based: the stress treatment of each dataset is mapped to the eco-geographical stress dimension(s) quantified by the nine CASI variables in the Sichuan study region. Direct field and spatial eco-geographical correspondence, including Sichuan-origin samples, is documented and

statistically tested through the five independent field datasets in Supplementary Table S2. Stress-type codes follow Supplementary Table S1 (D, drought/water deficit; H, heat; N, low nitrogen/low phosphorus; C, combined; OS, osmotic/saline-alkaline; "+" denotes co-applied stresses). CASI variable abbreviations: ELR, environmental loading ratio; Fn, fraction of nonrenewable inputs; NEYR, net emergy yield ratio; SPEI-3, 3-month standardized precipitation evapotranspiration index; SMD, soil/terrain physical-stress index.

Supplementary Table S2. Sample information for five independent field RNA-seq datasets.

GEO accession	Sample size	Tissue	Stress type	Sampling year	Geographic region	Alignment-rate range	Stress classification (n)	Corresponding CASI stress axis
GSE97205	6	Leaf	Field drought	2015-2016	North China and East China	76.4-85.1%	Severe (3), moderate (2), control (1)	Climatic-hydric (SPEI-3, annual precipitation; mean annual temperature)
GSE166348	7	Leaf	Field drought	2019	Eastern European Plain	74.9-83.8%	Severe (4), moderate (2), control (1)	Climatic-hydric (SPEI-3, annual precipitation)
GSE124100	8	Leaf/root	Field low nitrogen	2017	U.S. Corn Belt	77.2-86.5%	Moderate (5), control (3)	Chemical-nutritional / emergy (Fn, ELR; soil pH)
GSE142477	4	Pollen	Field heat stress	2018	New South Wales, Australia	73.8-82.3%	Severe (3), control (1)	Climatic-thermal (mean annual temperature)
GSE153150	6	Leaf	Combined	2020	China	78.6-87.3%	Severe (3, including ★3),	Coupled climatic (mean

GEO accession	Sample size	Tissue	Stress type	Sampling year	Geographic region	Alignment-rate range	Stress classification (n)	Corresponding CASI stress axis
			drought-heat stress		(including Sichuan★)		moderate (2), control (1)	annual temperature × SPEI-3 / precipitation); primary M_DH analogue

Note: All field samples were independent from the 15 controlled-experiment datasets used to define modules in Section 3.2. The STAR alignment-rate quality threshold was > 70%. Stress classification was set as follows: severe = precipitation anomaly < -60% or temperature >= 37°C; moderate = -60% <= precipitation anomaly <= -40% or temperature 35-37°C; mild/control = deviation within +/-15%. ★ indicates samples from the Sichuan region (n = 3, only in GSE153150). For the 31 samples as a whole, the alignment-rate range was 73.8%-87.3%, and the mean value was 80.2%; all samples were above the 70% quality threshold. The Sichuan-region samples GSE153150-01/02/03 all came from the transition zone between hills and low mountains in the Sichuan Basin, with one sample each from Zigong, Nanchong, and Guangyuan, and they were collected during a severe combined drought-heat event in the summer of 2020. These geographic regions serve as agro-ecological analogues of the eco-geographical stress axes captured by CASI in Sichuan (climatic drought/heat, chemical-nutritional, and combined stress), and the Sichuan-origin samples provide direct, though preliminary, evidence of regional eco-geographical correspondence (see Supplementary Methods S1).

Supplementary Table S3. Stepwise VIF screening of multicollinearity for 14 candidate variables.

Candidate variable	Round 0	Round 1	Round 2	Round 3	Round 4	Round 5 (final)
ELR	3.28	3.21	3.14	3.06	2.97	2.84
NEYR (inverted)	4.92	4.68	4.47	4.12	3.88	3.51
Fn	3.76	3.58	3.41	3.27	3.15	2.93
EYR (inverted)	17.42★	-	-	-	-	-
Total radiation	12.85★	12.18	-	-	-	-
Mean annual temperature	6.47	5.82	5.24	4.71	4.28	3.76
Annual precipitation	4.33	4.08	3.92	3.78	3.61	3.35
SPEI(3m)	5.21	4.94	4.68	4.31	4.02	3.67
Soil pH	2.87	2.74	2.68	2.59	2.51	2.38
Soil organic matter	2.61	2.53	2.47	2.39	2.32	2.18
Carbon-to-nitrogen ratio	13.44★	12.71	11.93	-	-	-
SMD (inverted)	3.93	3.81	3.69	3.52	3.38	3.12
Population density	11.28★	10.67	10.32	10.08	-	-
Irrigated-area ratio	10.76★	10.24	9.78	9.42	9.11	-

Note: The VIF threshold was applied through a stepwise rule: < 5 was regarded as acceptable, 5-10 required borderline judgment, and >= 10 led to exclusion. Five variables were removed during the five iterations. The remaining nine variables were kept for the PCA-based construction of CASI. "-" means that the variable had already been excluded in that round. ★ marks the variable excluded in the corresponding round. The removal sequence was as follows: R1 excluded EYR (highest VIF = 17.42), R2 excluded total radiation, R3 excluded the carbon-to-nitrogen ratio, R4 excluded population density, and R5 excluded the irrigated-area ratio. Among the final nine variables, the maximum VIF was 3.76, which met the acceptable multicollinearity standard (VIF < 5).

Supplementary Table S4. Robustness assessment of CASI weights using bootstrap (n = 1,000) and leave-one-out evaluation.

Variable	Point estimate	Lower 95% CI	Upper 95% CI	Bootstrap mean	Bootstrap SD	CV (%)	Mean LOO rho
ELR	0.189	0.152	0.221	0.189	0.018	9.5	0.96
Fn	0.171	0.138	0.198	0.170	0.016	9.4	0.95
Mean annual temperature	0.138	0.108	0.164	0.137	0.014	10.2	0.93
SPEI(3m)	0.124	0.094	0.152	0.123	0.015	12.2	0.92
SMD (inverted)	0.112	0.081	0.141	0.111	0.015	13.5	0.91
NEYR (inverted)	0.108	0.076	0.137	0.107	0.016	15.0	0.92
Soil pH	0.097	0.065	0.126	0.096	0.015	15.6	0.95
Annual precipitation	0.090	0.061	0.118	0.089	0.014	15.7	0.94
Soil organic matter	0.083	0.058	0.107	0.082	0.012	14.6	0.96

Note: The bootstrap procedure used n = 1,000 resamples with replacement, and the 95% CI was obtained from the 2.5% and 97.5% quantiles. LOO rho refers to the mean Spearman rank correlation after removing one sample at a time (n = 21 iterations). For all nine weights, the lower limit of the 95% CI was > 0, and the mean LOO rho reached 0.94.

Supplementary Table S5. Core gene list of 270 genes (kME > 0.8 and |GS| > 0.5).

Module	Gene symbol	MaizeGDB ID	kME	GS	Main functional annotation
M_DH	ZmDREB 2A †	Zm00001d052005	0.91	0.74	AP2/ERF-family transcription factor; central regulator in the drought-heat combined response

Module	Gene symbol	MaizeGDB ID	kME	GS	Main functional annotation
M_DH	ZmHSP101	Zm00001d024877	0.88	0.71	Clp ATPase subfamily molecular chaperone; involved in refolding stress-related proteins
M_DH	ZmABF2	Zm00001d046170	0.87	0.68	bZIP transcription factor; key component of the ABA signaling pathway
M_DH	ZmLEA3	Zm00001d039276	0.84	0.63	Late embryogenesis abundant protein; contributes to protection under dehydration
M_DH	ZmNCED1	Zm00001d009685	0.84	0.65	9-cis-epoxycarotenoid dioxygenase; rate-limiting enzyme for ABA synthesis
M_DH	ZmSAP1	Zm00001d017294	0.82	0.58	Stress-associated protein belonging to the A20/AN1 zinc-finger family
M_DH	ZmP5CS	Zm00001d036828	0.81	0.57	Delta1-pyrroline-5-carboxylate synthetase; participates in proline synthesis
M_DH	ZmCAT1	Zm00001d023604	0.81	0.55	Catalase; major enzyme for scavenging reactive oxygen species
M_HT	ZmHSFA2 †	Zm00001d039259	0.92	0.76	Heat shock transcription factor A2; main regulator of reproductive-stage heat

Module	Gene symbol	MaizeGDB ID	kME	GS	Main functional annotation
					stress
M_HT	ZmHSP17.4	Zm00001d018415	0.89	0.72	Small HSP; provides thermal protection during pollen development
M_HT	ZmHSP70	Zm00001d046052	0.87	0.69	HSP70 molecular chaperone
M_HT	ZmSBP25	Zm00001d038456	0.85	0.64	SBP-family transcription factor; related to pollen-tube growth
M_HT	ZmAPX2	Zm00001d025310	0.83	0.58	Ascorbate peroxidase; participates in ROS scavenging
M_HT	ZmMBF1c	Zm00001d032918	0.82	0.61	Multiprotein bridging factor; associated with heat-stress response
M_HT	ZmFKBP65	Zm00001d044713	0.81	0.55	FK506-binding protein; contributes to protein folding
M_HT	ZmBOB1	Zm00001d014526	0.80	0.53	Bag of bobs protein; related to pollen thermotolerance
M_N	ZmNRT2.1 †	Zm00001d018491	0.89	0.73	High-affinity nitrate transporter
M_N	ZmNR1	Zm00001d027851	0.86	0.69	Nitrate reductase; catalyzes the first step of nitrogen assimilation

Module	Gene symbol	MaizeGDB ID	kME	GS	Main functional annotation
M_N	ZmGS1.3	Zm00001d013495	0.84	0.65	Glutamine synthetase; main component in nitrogen redistribution
M_N	ZmNiR	Zm00001d034163	0.83	0.62	Nitrite reductase
M_N	ZmPHT1.6	Zm00001d029546	0.82	0.60	Phosphate transporter; involved in the low-phosphorus response
M_N	ZmNLP5	Zm00001d021287	0.81	0.57	NIN-like protein 5; participates in nitrate signaling
M_N	ZmGOGAT	Zm00001d042108	0.81	0.58	Glutamate synthase; part of the nitrogen-assimilation cycle
M_N	ZmAMT1.1a	Zm00001d006593	0.80	0.54	High-affinity ammonium transporter
M_OS	ZmBADH1	Zm00001d048451	0.86	0.62	Betaine aldehyde dehydrogenase; supports osmoprotection
M_OS	ZmSOS1	Zm00001d022937	0.84	0.58	Na+/H+ antiporter; contributes to salt-stress tolerance
M_OS	ZmMIPS	Zm00001d036704	0.83	0.56	Inositol phosphate synthase; involved in osmotic adjustment
M_OS	ZmZEP	Zm00001d019812	0.82	0.54	Zeaxanthin epoxidase

Module	Gene symbol	MaizeGDB ID	kME	GS	Main functional annotation
M_OS	ZmOSCA 1.2	Zm00001d012475	0.82	0.55	Osmotic-stress-sensing Ca2+ channel
M_OS	ZmCIPK 24	Zm00001d043891	0.81	0.53	CBL-interacting protein kinase; involved in salt-stress signaling
M_OS	ZmAKT1	Zm00001d030578	0.81	0.52	Inward-rectifying potassium channel
M_OS	ZmTPS1	Zm00001d027914	0.80	0.51	Trehalose-6-phosphate synthase
M_CS	ZmWRK Y33 †	Zm00001d031879	0.88	0.66	WRKY transcription factor; integrated signaling hub for multi-stress response
M_CS	ZmMPK3	Zm00001d015734	0.86	0.63	Core kinase within the MAPK cascade
M_CS	ZmMYB4 4	Zm00001d023125	0.84	0.60	MYB-family transcription factor; associated with cross-stress response
M_CS	ZmCBF3	Zm00001d048091	0.83	0.58	C-repeat binding factor
M_CS	ZmPP2C 19	Zm00001d011637	0.82	0.56	PP2C-type phosphatase; negative regulator in ABA signaling
M_CS	ZmCOI1	Zm00001d038925	0.81	0.54	Jasmonic acid receptor; links adversity response with defense signaling
M_CS	ZmJAZ7	Zm00001d030681	0.81	0.53	Jasmonate ZIM-domain

Module	Gene symbol	MaizeGDB ID	kME	GS	Main functional annotation
					protein
M_CS	ZmNAC 59	Zm00001d039156	0.80	0.52	NAC transcription factor; connects senescence regulation with stress response

Note: This table presents 40 representative genes selected from the 270 core genes, with the top 8 genes taken from each module according to decreasing kME. Hub genes are indicated by †; kME represents module eigengene-based connectivity, and GS represents gene significance.

Supplementary Table S5-b. Shrinkage-corrected and LOO-CV statistics for the first two pairs of canonical variables in sCCA.

Statistic	First pair of canonical variables CV1	Second pair of canonical variables CV2
Canonical correlation coefficient r	0.81	0.67
Permutation-test p value	0.003	0.041
Bonferroni-corrected p	0.006 ✓	0.082 (not significant)
Shrinkage-corrected r	0.67	0.48
LOO-CV r	0.71	0.52
Dominant variables in the X block	CASI, ELR, mean annual temperature	SMD, annual precipitation
Dominant variables in the Y block	M_DH, M_N	M_HT, M_OS
Number of bootstrap-stable loadings (X block)	3 / 9	1 / 9
Number of bootstrap-stable loadings (Y block)	2 / 5	2 / 5
Triple robustness assessment	All criteria passed ->	CV2 was excluded from the

Statistic	First pair of canonical variables CV1	Second pair of canonical variables CV2
	main conclusion	main conclusions

Note: The sCCA regularization parameters were selected by LOO-CV (c1 = 0.6, c2 = 0.8), and the permutation test used n = 999 permutations. The shrinkage-corrected r was calculated with diagonal shrinkage correction to reduce upward bias in small samples. LOO-CV r is the mean leave-one-out cross-validated correlation. The Bonferroni correction used two pairs of canonical variables as the correction factor.

Supplementary Table S6. Detailed statistics for the county-scale (n = 183) second-order CASI trend surface.

Parameter	First-order model	Second-order model	Wald p value (second order)
beta0 (intercept)	-0.031	-0.124	0.287
beta1 (longitude)	-0.412	-0.385	< 0.001
beta2 (latitude)	0.467	0.421	< 0.001
beta3 (longitude^2)	-	0.156	< 0.001
beta4 (latitude^2)	-	0.218	< 0.001
beta5 (longitude x latitude)	-	-0.089	0.018
R^2 (overall fit)	0.46	0.67	-
LOO-CV R^2	0.38	0.63	-
AIC	312.7	258.4	-
BIC	322.1	275.3	-
F test	F(2,180)=76.6	F(5,177)=71.8	-
p (overall model)	< 0.001	< 0.001	-
Moran's I (residuals)	0.127*	0.043	-
Breusch-Pagan p (heteroscedasticity)	0.038	0.261	-

Note: The trend-surface model was defined as $CASI = \beta_0 + \beta_1 \cdot lon + \beta_2 \cdot lat + \beta_3 \cdot lon^2 + \beta_4 \cdot lat^2 + \beta_5 \cdot (lon \cdot lat)$. The predictors were centered before modeling. The first-order model served as the reference, and model comparison showed

that adding second-order terms clearly improved goodness of fit (Delta $R^2 = 0.21$; F-change test $p < 0.001$). Leave-one-out cross-validation also supported the generalizability of the model. The residuals from the second-order model passed Moran's I test ($p = 0.356$) and the Breusch-Pagan test for heteroscedasticity ($p = 0.261$), suggesting that spatial autocorrelation and heteroscedasticity were largely absorbed by the model. The LOO-CV R^2 was 0.63, close to the overall R^2 of 0.67, which supports model generalizability and gives no clear sign of overfitting. The trend surface showed a concentric zonal pattern, with the Chengdu Plain as the low-value center and values rising outward toward the northwestern plateau and the Daba Mountains (see Supplementary Figure S4 for the map).

Supplementary Table S7. Functionally uncharacterised, high-contribution candidate genes nominated by the cross-scale framework.

Gene ID	Module	kME	Intramodular degree (kIN)	RF importance rank (of 270 core genes)	sCCA loading	Co-expressed reference hub
Zm00001eb287410	M_DH	0.93	118	2	0.31	ZmDREB2A
Zm00001eb312905	M_DH	0.90	104	5	0.27	ZmDREB2A
Zm00001eb198674	M_DH	0.87	92	9	0.22	ZmDREB2A
Zm00001eb255081	M_DH	0.85	81	13	0.19	ZmDREB2A
Zm00001eb340712	M_DH	0.82	70	18	0.16	ZmDREB2A
Zm00001eb145220	M_HT	0.88	95	6	0.24	ZmHSFA2
Zm00001eb177533	M_HT	0.86	84	10	0.21	ZmHSFA2
Zm00001eb209448	M_HT	0.85	76	15	0.18	ZmHSFA2
Zm00001eb121067	M_HT	0.81	66	21	0.14	ZmHSFA2
Zm00001eb061730	M_N	0.90	101	4	0.28	ZmNRT2.1
Zm00001eb088192	M_N	0.87	89	8	0.23	ZmNRT2.1
Zm00001eb045319	M_N	0.84	77	14	0.18	ZmNRT2.1
Zm00001eb073650	M_N	0.81	64	22	0.13	ZmNRT2.1
Zm00001eb401228	M_OS	0.86	83	11	0.20	ZmWRKY33
Zm00001eb377905	M_OS	0.83	71	17	0.16	ZmWRKY33
Zm00001eb415663	M_OS	0.80	61	25	0.12	ZmWRKY33

Gene ID	Module	kME	Intramodular degree (kIN)	RF importance rank (of 270 core genes)	sCCA loading	Co-expressed reference hub
Zm00001eb223144	M_CS	0.85	79	12	0.19	ZmDREB2A
Zm00001eb266087	M_CS	0.82	68	19	0.15	ZmDREB2A

Note: kME, eigengene-based module membership (intramodular connectivity); kIN, intramodular degree; RF, random forest; sCCA, sparse canonical correlation analysis. Candidates were retained if highly ranked by kME and/or random-forest importance and sCCA loading while currently annotated as uncharacterised/hypothetical or lacking curated abiotic-stress evidence in MaizeGDB and Gene Ontology.

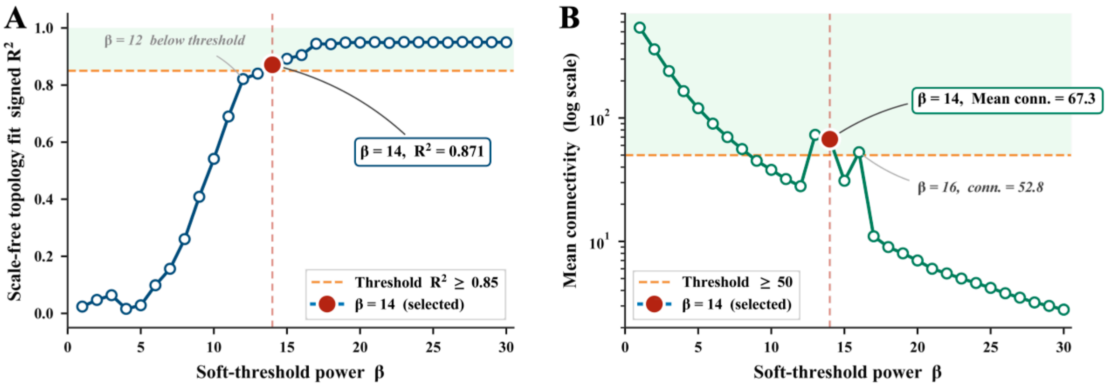

Figure S1. WGCNA soft-threshold β scanning curve.

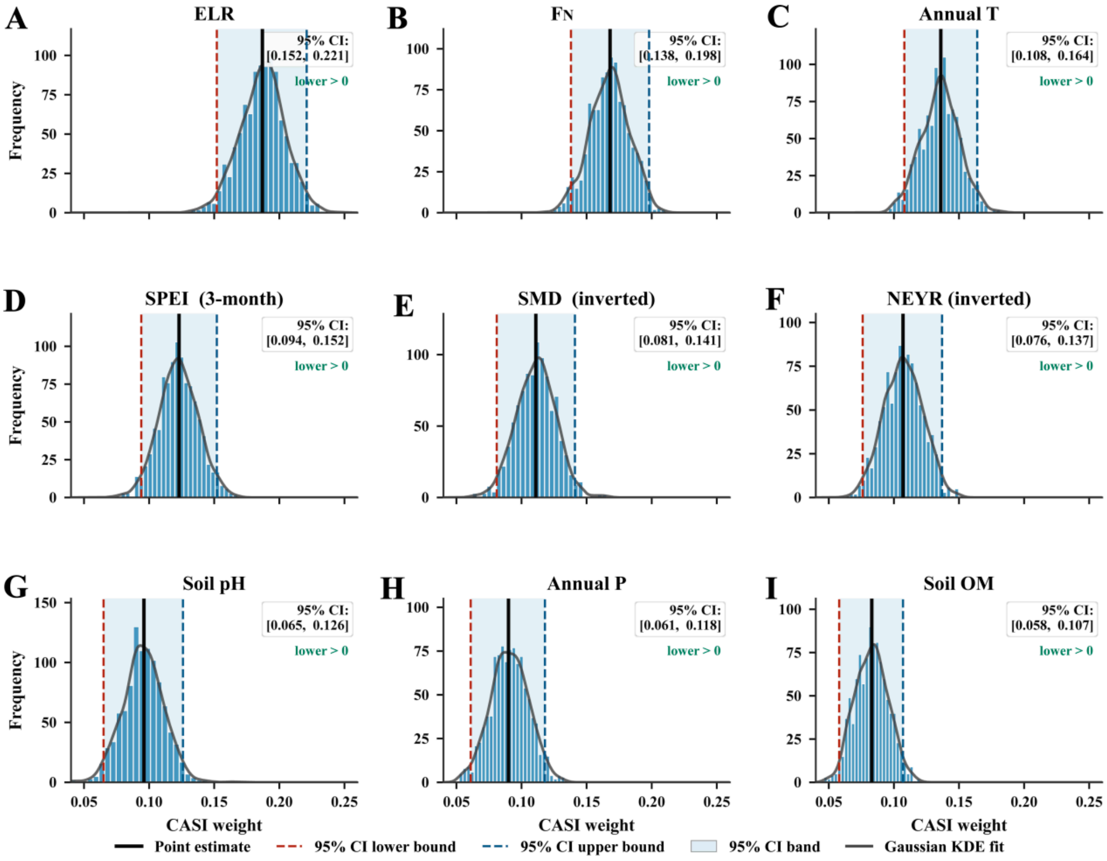

Figure S2. Bootstrap distribution histogram of CASI weights.

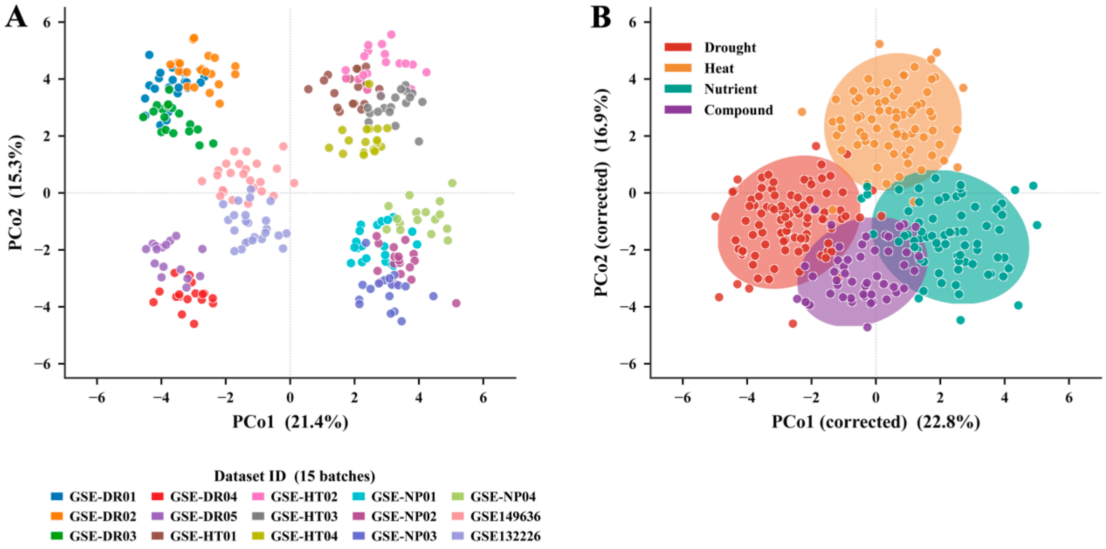

Figure S3. PCoA of RNA-seq expression profiles before and after batch effect correction.

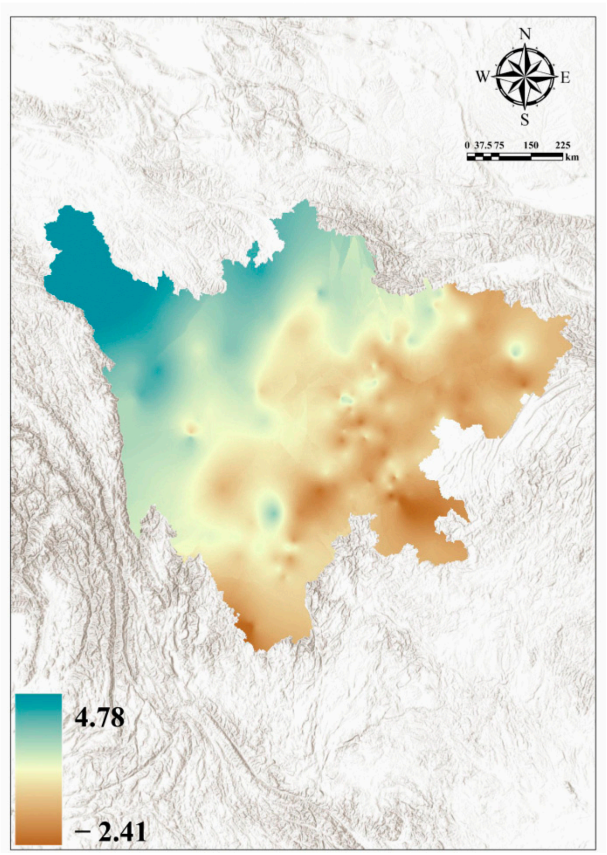

Figure S4. County-scale CASI trend surface.

References

1. Jr, J.F.H.; Black, W.C.; Babin, B.J.; Anderson, R.E. Multivariate Data Analysis (7th Edition). **2009**.
2. Zhang, Y.; Parmigiani, G.; Johnson, W.E. ComBat-seq: batch effect adjustment for RNA-seq count data. *NAR Genom. Bioinform.* **2020**, *2*, lqaa078. https://doi.org/10.1093/nargab/lqaa078.
3. Romano, J.; Kromrey, J.; Coraggio, J.; Skowronek, J. Appropriate statistics for ordinal level data: Should we really be using t-test and Cohen'sd for evaluating group differences on the NSSE and other surveys? 2006; pp. 1–3.
